# The Dengue Virus NS5 Protein Intrudes in the Cellular Spliceosome and Modulates Splicing

**DOI:** 10.1371/journal.ppat.1005841

**Published:** 2016-08-30

**Authors:** Federico A. De Maio, Guillermo Risso, Nestor G. Iglesias, Priya Shah, Berta Pozzi, Leopoldo G. Gebhard, Pablo Mammi, Estefania Mancini, Marcelo J. Yanovsky, Raul Andino, Nevan Krogan, Anabella Srebrow, Andrea V. Gamarnik

**Affiliations:** 1 Fundación Instituto Leloir-CONICET, Buenos Aires, Argentina; 2 Instituto de Fisiología, Biología Molecular y Neurociencias (IFIBYNE, UBA-CONICET), Departamento de Fisiología, Biología Molecular y Celular, Facultad de Ciencias Exactas y Naturales, Universidad de Buenos Aires; 3 Department of Microbiology and Immunology, University of California, San Francisco, San Francisco, California, United States of America; 4 Department of Cellular and Molecular Pharmacology, University of California, San Francisco, California, United States of America; NIH, UNITED STATES

## Abstract

Dengue virus NS5 protein plays multiple functions in the cytoplasm of infected cells, enabling viral RNA replication and counteracting host antiviral responses. Here, we demonstrate a novel function of NS5 in the nucleus where it interferes with cellular splicing. Using global proteomic analysis of infected cells together with functional studies, we found that NS5 binds spliceosome complexes and modulates endogenous splicing as well as minigene-derived alternative splicing patterns. In particular, we show that NS5 alone, or in the context of viral infection, interacts with core components of the U5 snRNP particle, CD2BP2 and DDX23, alters the inclusion/exclusion ratio of alternative splicing events, and changes mRNA isoform abundance of known antiviral factors. Interestingly, a genome wide transcriptome analysis, using recently developed bioinformatics tools, revealed an increase of intron retention upon dengue virus infection, and viral replication was improved by silencing specific U5 components. Different mechanistic studies indicate that binding of NS5 to the spliceosome reduces the efficiency of pre-mRNA processing, independently of NS5 enzymatic activities. We propose that NS5 binding to U5 snRNP proteins hijacks the splicing machinery resulting in a less restrictive environment for viral replication.

## Author Summary

Mapping host-pathogen interactions has proven fundamental for understanding how viruses manipulate host machinery and how cellular processes are regulated during infection. Dengue virus poses a major threat to public health: two-thirds of the world’s population is now at risk from infection by this mosquito-borne virus. In this work, using a global proteomic approach in the context of viral infections with tagged dengue viruses, we constructed a comprehensive protein-protein interaction map of the multifunctional NS5 viral protein. NS5 is central for viral RNA replication and for immune evasion. Our studies revealed the interaction of NS5 with core components of the splicing machinery, specifically with proteins of the U5 small nuclear ribonucleoprotein particle, and that viral infection reduces splicing efficiency. Mechanistic studies analyzing endogenous splicing events and in vitro splicing assays indicated that NS5 binds active spliceosomes and reduces the efficiency of pre-mRNA processing. Our results provide a new function of the dengue virus NS5 protein and support a model in which manipulation of specific splicing components favors viral infection.

## Introduction

Dengue virus (DENV) is currently the most important human viral pathogen transmitted by insects. It is responsible for about 390 million infections worldwide every year [1]. In spite of this great burden, vaccines and specific antivirals remain elusive. In fact, a steady increase in the number of infections is being registered in the last years (http://apps.who.int/iris/bitstream/10665/75303/1/9789241504034_eng.pdf?ua=1). DENV belongs to the Flavivirus genus in the Flaviviridae family, together with a large number of emerging and re-emerging human pathogens that cause fevers and encephalitis, such as West Nile virus, Japanese encephalitis virus and Zika virus [2,3].

Like in other RNA viruses, the DENV genome encodes a limited set of proteins, but relies on the host machinery for productive replication. During infection, viral components subvert cellular processes, remodeling intracellular membranes, changing host metabolic routes and blocking innate antiviral responses [4,5]. These changes in the cellular environment are the result of an intimate host-virus interaction and co-evolution. Although in the last decade a great deal has been learned about the DENV biology, the intricate network of viral-host interactions that provide the appropriate setting for viral replication is largely unknown.

Global mapping of protein-protein interactions through systematic overexpression of viral proteins and proteomic studies have recently identified complete cellular pathways harnessed by HIV and HCV infection [6–8]. Moreover, the generation of recombinant viruses able to replicate expressing tagged viral proteins allowed identification of protein complexes in the context of measles, influenza and HIV infections [9–11]. A technical limitation for this kind of proteomic approach is the feasibility to design recombinant viruses that tolerate the addition of tags. Here, we developed a tool for proteomic studies by incorporating purification tags in fully functioning DENV, and generated affinity purification-mass spectrometry data from infected human cells focusing on the viral protein NS5.

NS5 is the largest viral protein, bearing multiple enzymatic activities and functions during infection. It bears the RNA dependent RNA polymerase and methyl transferase activities, which are fundamental for viral genome amplification [12–18]. In addition, NS5 interacts with different host proteins to counteract the IFN-α mediated antiviral response through STAT2 degradation [19–21]. Although viral RNA replication takes place in the cytoplasm of the infected cell, NS5 distributes between the cytoplasm and the nucleus. Intriguingly, for certain DENV serotypes, the majority of NS5 accumulates in the nucleus [22–26]. The importance of this NS5 subcellular distribution remains unsolved and could be related to an auxiliary function that has not yet been elucidated. In order to identify new functions of NS5, we conducted an unbiased proteomic study and constructed a map of physical interactions between NS5 and host proteins in the context of a productive DENV infection. These studies revealed the presence of NS5 in complex with core components of the cellular splicing machinery.

The process of precursor mRNA (pre-mRNA) splicing involves the removal of introns and the precise joining of exons, which results in mature eukaryotic mRNAs [27]. The cellular splicing machinery, the spliceosome, is composed of five uridine-rich small nuclear ribonucleoprotein particles (U snRNPs) known as U1, U2, U4, U5 and U6 snRNPs and non-snRNP associated proteins. Each snRNP comprises a small RNA molecule (snRNA), a common set of proteins (Sm or SMN) and a number of particle-specific proteins [28]. The exon and intron definition depends on information present in the pre-mRNA: 5’ and 3’ splice sites (SS), branch sites (BS) as well as intronic or exonic enhancers and silencers that recruit regulatory proteins that modulate assembly and disassembly of spliceosomal complexes. The splicing machinery is dynamic and exceptionally flexible, allowing fine regulation of gene expression and protein function [27]. Relevant for our studies, modulation of splicing has been shown to be a regulatory mechanism for balancing the host antiviral response [29,30]. In this regard, a growing number of pathways that link viral infection, antiviral responses and splicing regulation are being considered as emerging antiviral evasion mechanisms [29–31].

Here, we identified a novel function of DENV NS5 in modulating cellular splicing. NS5 alone or in the context of viral infection binds to CD2BP2 and DDX23, core components of U5 snRNP, and incorporates in active spliceosomes. Analysis of endogenous splicing events showed that DENV infection affects splicing efficiency and alters cellular splicing patterns. Mechanistic studies using in vitro splicing assays and reporter minigenes in cultured cells indicate that NS5 interferes with pre-mRNA processing. Importantly, depletion of CD2BP2, DDX23 or EFTUD2 by RNA interference (RNAi) increases the efficiency of DENV infection, providing evidence for how NS5 works in promoting viral replication independently of its canonical enzymatic activities.

## Results

### Mapping physical interactions of DENV NS5 with host proteins in infected cells

The DENV NS5 protein plays multiple functions during viral infection, likely by interacting with different cellular components. In order to investigate physical interactions of NS5 with cellular proteins during infection, we undertook an unbiased proteomic approach. We used an affinity-purification mass spectroscopy (AP-MS) strategy in the context of an infectious virus carrying a tagged NS5 protein. To this end, we developed recombinant DENVs encoding modified NS5 proteins. In contrast to over-expressing the individual protein, this approach facilitates identification of protein-protein interactions in a cellular environment that has been conditioned by the viral infection.

**Fig 1:**
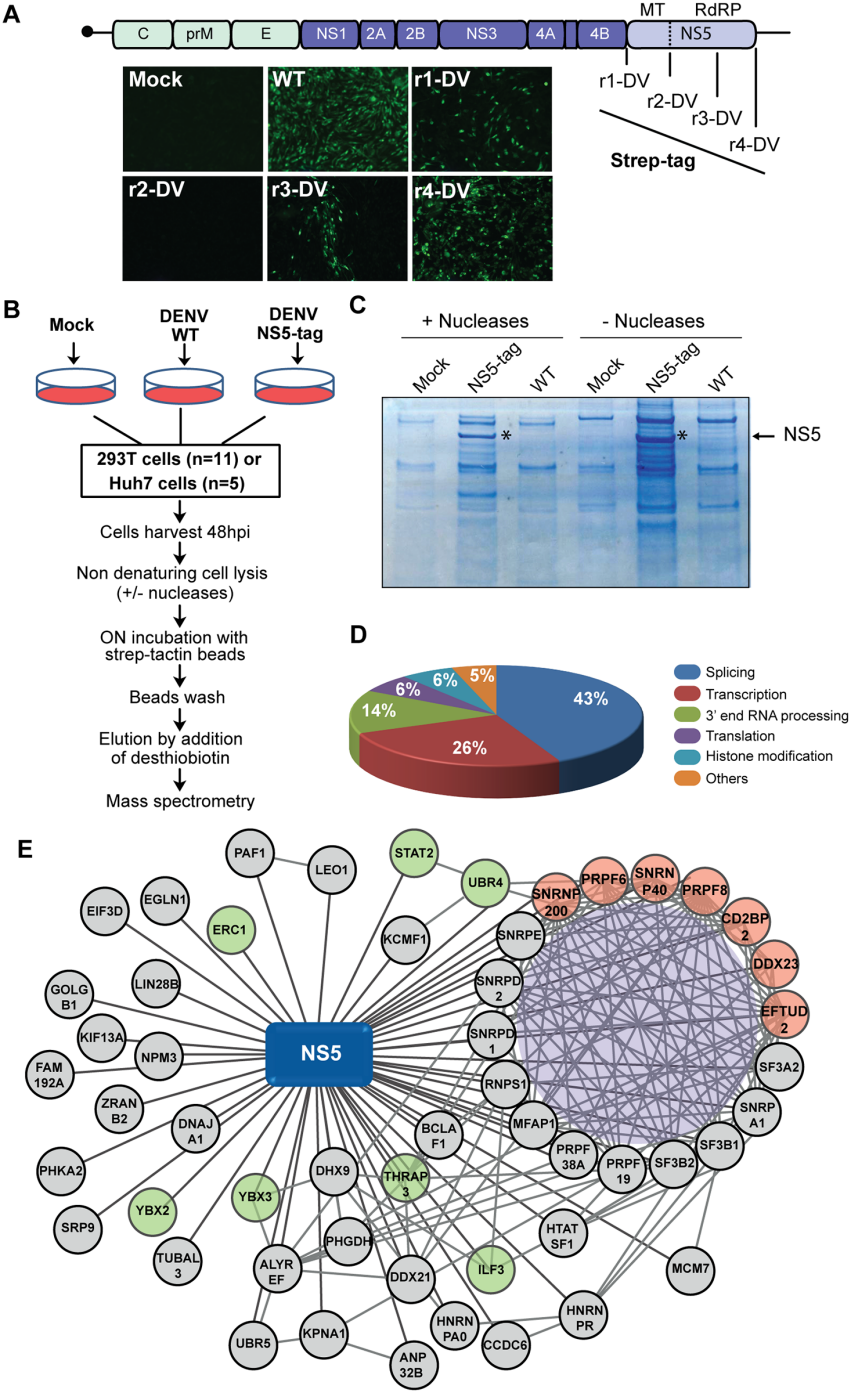
Interaction network of DENV NS5 and human proteins from infected cells. (A) Construction of recombinant DENVs. Viruses with Strep-tag fused to NS5 in different positions: N-terminus (r1-DV), inter-domain linker (r2-DV), low conserved loop region (r3-DV) and C-terminus (r4-DV) are shown. Immunofluorescence assays after RNA transfection using specific anti-E antibodies show replication of each virus. (B) Flow chart of experimental procedure for NS5-containing protein complex purification. Human cells (HEK 293T or Huh7) were mock infected, DENV WT infected, or DENV NS5-tag infected in parallel. (C) Coomassie blue staining showing the recovery of NS5 binding partners. (D) Results of the mass spectrometry analysis, the major functional categories of host proteins identified as NS5 interactors are depicted. (E) NS5-host proteins interaction network. Lines depict interactions detected in this study between NS5 and human proteins. Also, interaction between human proteins extracted from database (http://string-db.org) is shown. Components of the spliceosomal U5 snRNP are shown in red. Previously reported NS5 interactors are indicated in green.

Using the 3D structure of NS5 [13], four different sites were selected to incorporate a purification tag (2 X Strep) in the DENV2 16681 full length clone. The replication capacities of recombinant viruses were compared with that of the WT by immunofluorescence (IF) assays after RNA transfection (Fig 1A). Only one of these viruses (r4-DV) retained a high replication capacity. A comparative analysis of the WT and r4-DV by IF as a function of the time and growth curves indicated similar replication of the two viruses (S1 Fig). Therefore, the r4-DV was employed as the tool to establish a protocol for NS5-host protein complex isolation and purification (Fig 1B). The pull-down was conducted under mild (non-denaturing) conditions, in order to recover proteins that interact either directly or indirectly with NS5.

Each NS5 purification experiment included three different treatments with the corresponding cell extracts then processed in identical conditions: a) mock infected, b) DENV WT infected, and c) DENV NS5-tag infected (Fig 1B). This experiment was repeated sixteen times using two different cell lines, in the presence and absence of nucleases. Eluates were examined by western blotting, coomassie blue and silver staining and submitted to mass spectrometry analysis. It is worth noting that a reduction in the amount of purified NS5 binders was observed if comparing cell lysates treated or not with nucleases, suggesting the presence of nucleic acid-mediated binders (Fig 1C). Based on a total of 48 samples analyzed, we identified 362 cellular proteins as potential NS5 interactors. This list was shortened to 53 candidate proteins after removing any candidates that i) appeared in negative control experiments (untagged virus or uninfected cells), ii) were not reproducible in >50% of the experiments, and iii) were promiscuous protein binders based on databases of over-expression studies [32]. In this regard, proteomic data from strep tagged viral proteins (capsid, NS3 and NS4B) were also used as controls to confirm specificity (S1 Table). The set of 53 proteins exhibited an important enrichment in proteins involved in transcription and RNA metabolism. In particular, about 40% of these proteins were splicing related factors, with many of them known as core spliceosomal components (Fig 1D and 1E). Among the splicing proteins detected as NS5 interactors, the most represented complex was the U5 snRNP, for which seven of the eight specific core components were present, suggesting a strong interaction with this particular snRNP (Fig 1E). It is important to highlight the consistent appearance of two members of this particle CD2BP2 and the helicase DDX23. In addition, previously identified partners of NS5 such as STAT2 and UBR4, and the splicing proteins PRPF8 and SNRNP200 were also detected [21,33].

In summary, we constructed a comprehensive NS5-host protein interactome map in the context of a DENV infection in human cells.

### The viral protein NS5 associates with active spliceosomes

**Fig 2:**
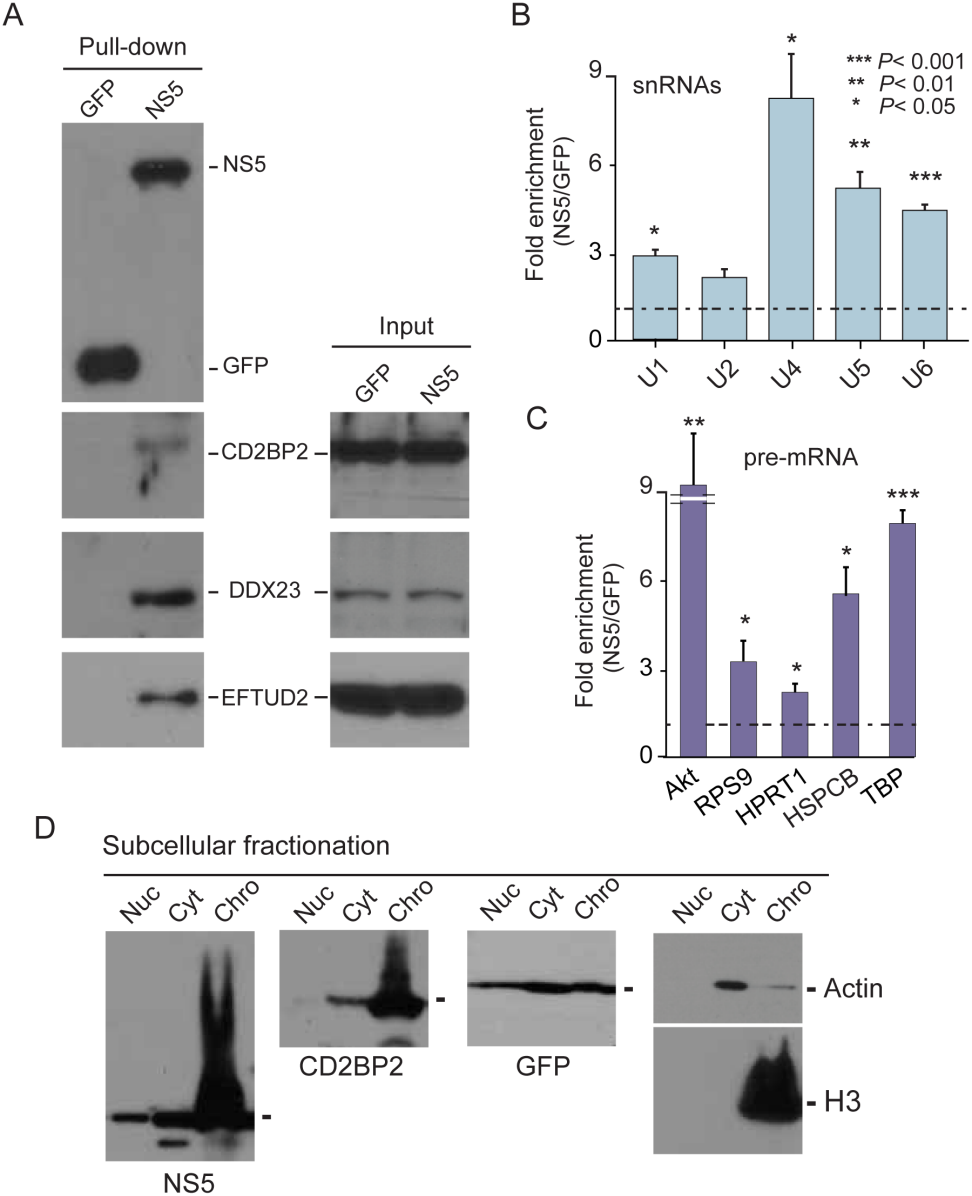
NS5 interacts with splicing proteins in the context of active spliceosomes. (A) NS5 binds CD2BP2, DDX23, and EFTUD2. Pull down assays and western blot analysis of indicated proteins are shown. (B) NS5 binds to assembled snRNPs containing the U1, U2, U4, U5, and U6 snRNAs. (C) Detection of indicated pre-mRNAs in the NS5 associated complexes. (D) Subcellular fractionation showing that NS5 accumulates in the insoluble chromatin fraction, similar to CD2BP2, a U5 snRNP component (Nuc: nucleoplasm, Cyt: cytoplasm, Chro: chromatin). Fractionation of cells expressing GFP is also shown as control, indicating that this protein distributes along different cellular compartments.

To further examine NS5 interaction with core U5 snRNP components, we evaluated interactions with CD2BP2, DDX23, and EFTUD2. The mature tagged viral protein was individually expressed in cultured human cells and extracts were used for non-denaturing pull down assays and western blot analysis. As a control, the same experimental procedure was carried out with a GFP expression vector. NS5 and GFP were expressed and purified with similar efficiencies (Fig 2A). In contrast, co-precipitation of CD2BP2, DDX23, and EFTUD2 was only observed with NS5 (Fig 2A, left panels), while the input content of the splicing factors in cells expressing GFP or NS5 was comparable (Fig 2A, right panels). To examine whether NS5 binds to assembled complexes, we performed RIP analysis and evaluated the presence of the RNA component (snRNAs) of different snRNPs, since the presence of these molecules is indicative of fully assembled ribonucleoprotein particles. RNA isolated from pull-down experiments obtained from cells expressing NS5 or GFP was used for quantification of different snRNAs by RT-qPCR. Significant enrichment of U1, U2, U4, U5, and U6 RNAs from 2 to 9 fold was observed in the complexes purified with NS5 in relation to that with GFP (Fig 2B). We next explored whether NS5 binds to active spliceosomes by measuring the presence of immature transcripts (pre-mRNAs) that are being spliced out in complex with NS5. To this end, we evaluated the association of different pre-mRNAs including housekeeping or highly expressed endogenous genes (HSPCB, Akt, RPS9, HPRT1 and TBP) to either NS5 or GFP-containing complexes. We found a significant enrichment of these pre-mRNAs in complexes with the viral protein NS5 compared to GFP (Fig 2C). To further confirm this observation, we analyzed the subcellular localization of NS5. Considering that active spliceosomes are mainly associated with chromatin [34], we carried out subcellular fractionation and evaluated the presence of NS5 in the cytoplasm, nucleoplasm and chromatin fractions. As mentioned above, the viral protein NS5 has been previously demonstrated to translocate into the nucleus [22–26]. In this analysis, an evident accumulation of NS5 in the insoluble chromatin fraction was observed together with histone H3 and the U5 snRNP component CD2BP2 (Fig 2D). Taken together, these results provide evidence that NS5 interacts with splicing proteins while they are integrated into assembled snRNPs as part of active spliceosomes.

### DENV infection changes splicing of endogenous mRNAs

After confirming that the viral protein NS5 interacts with components of the cellular splicing machinery, both by individual expression and in the context of viral infection, we hypothesize that NS5 modulates host splicing. To assess the functional impact of this interaction on cellular splicing patterns, we first evaluated a number of well-characterized endogenous alternative splicing events in infected or mock infected cells.

**Fig 3:**
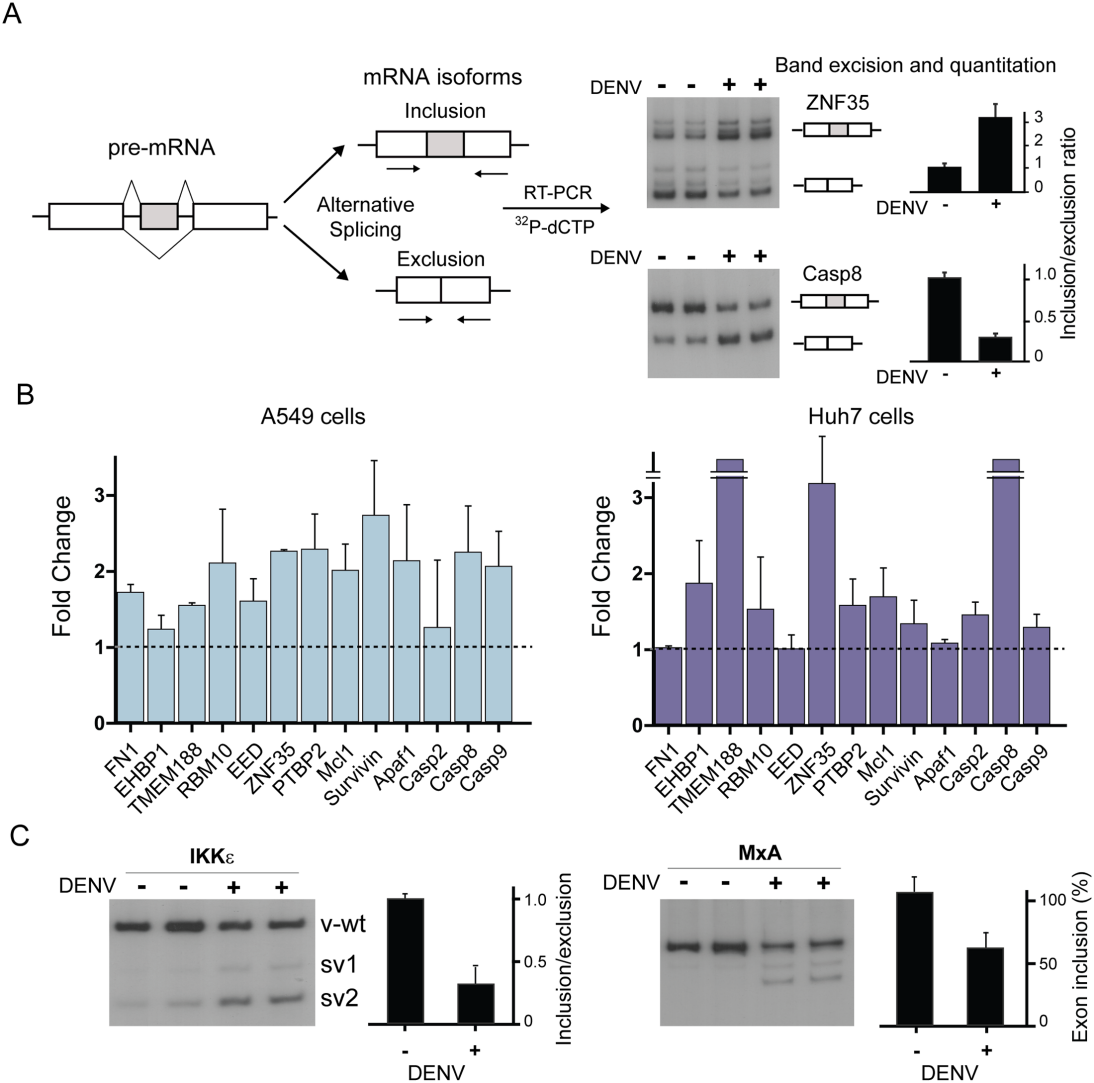
DENV infection modifies the splicing landscape of host cells. (A) Strategy to detect and quantify alternative splicing isoforms in mock infected or DENV infected cells is depicted on the left. Representative autoradiographs and quantification of the inclusion/exclusion ratio for two alternative exon cassette events are shown (duplicates, mean ± SD). (B) Fold-change of inclusion/exclusion ratios for DENV or mock infected cells. A battery of endogenous alternative exon-cassette events (duplicates, mean ± SD) in A549 and Huh7 cells is shown. (C) DENV-induced changes in the splicing pattern of IKKε and MxA mRNAs. Quantification of alternative splicing events is shown for each case.

For each particular splicing event, we analyzed the relative abundance of two mRNA isoforms (lacking or containing an alternative exon cassette) by RT-PCR. The radiolabeled PCR products were separated by gel electrophoresis, excised from the gel and quantified, as depicted in Fig 3A. The inclusion/exclusion ratios can either increase or decrease in different cellular conditions, also depending on the specific splicing event analyzed. Examples of these two situations in DENV infected cells are shown in Fig 3A and S2 Fig (ZNF35 and Casp8). In both cases, the relative abundance of the different isoforms was significantly modified by viral infection. This analysis was extended to a battery of alternative splicing events in two human cell lines (Fig 3B). The fold change of isoforms in infected versus uninfected cells showed that around 40% of the analyzed events were substantially modified during infection. These results provide evidence of an impact of DENV2 infection on cellular splicing. Because the nuclear localization of NS5 differs among DENV serotypes [22,24], we also examined a subset of splicing events upon DENV4 infection (S3 Fig). The results suggest that the two viruses modulate splicing to a different extent, providing evidence of a possible serotype specific alteration of splicing.

It has been previously reported that modulation of alternative splicing is a mechanism to regulate the balance of the cellular antiviral state [30,35–38]. For instance, alternative splicing of IKKε results in different exon-skipping isoforms with one variant that retains full antiviral activity (v-wt) and two variants (v1 and v2) that yield a dominant negative or partially inactive protein [39].

To extend our observation, we examined splicing variants of IKKε and MxA in DENV infected or mock infected cells. In the case of IKKε, an increased abundance of sv1 and sv2 relative to the v-wt was observed, suggesting the expression of dominant negative forms of IKKε during DENV infection (Fig 3C). Similarly, in the case of MxA, the appearance of exclusion isoforms was observed concurrently with a reduction in the amount of the canonical (inclusion) isoform (Fig 3C). This resembles a shift in the splicing pattern previously described for herpes virus infection, in which an MxA isoform supports instead of restricting viral infection [40]. Together, these observations suggest a model in which DENV infection alters cellular splicing patterns leading to changes in isoform abundance of antiviral factors, which could facilitate viral replication.

### Direct impact of NS5 on cellular splicing

The viral protein NS5 physically interacts with the spliceosome when individually expressed in human cells, and DENV infection causes a pronounced alteration of endogenous cellular splicing. Thus, we speculate that NS5 is, at least in part, responsible for splicing modulation during viral infection. To examine this possibility, splicing was monitored by analyzing mRNA isoforms derived from splicing reporter minigenes co-transfected with expression vectors for different variants of NS5 or control proteins. The reporter minigenes are cloned gene fragments that include the splicing event of interest, in particular, an alternative exon and the flanking intronic regions, downstream of a constitutive promoter. Transcription from this promoter generates minigene-derived pre-mRNA molecules, which are canonically processed to render distinguishable mature products. These reporters have been widely used to characterize cis-acting elements and trans-acting splicing modulators [41].

**Fig 4:**
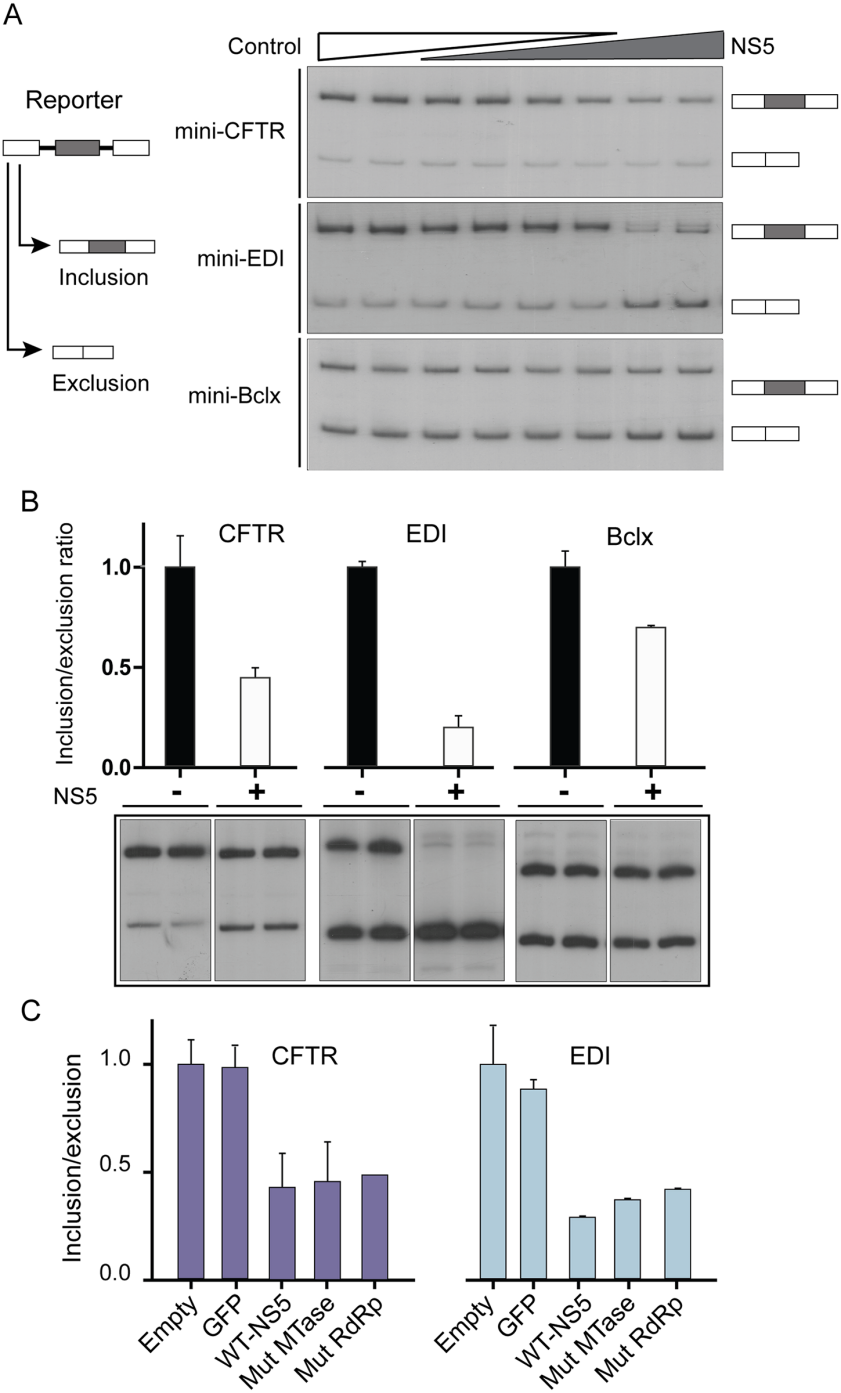
NS5 alone interferes with alternative splicing. (A) NS5 shows a dose dependent effect on the splicing reporter minigenes CFTR, EDI, and Bclx. Radiolabeled amplification products corresponding to different splicing isoforms derived from the indicated minigenes are shown. (B) Quantitative analysis of the changes induced by NS5 (0.9 μg of plasmid). (C) Expression of NS5 mutants with impaired methyltransferase (Mut-MTase) or polymerase (Mut-RdRp) enzymatic activity also alters alternative splicing of reporter genes.

In our experimental setting, we used three different splicing reporter minigenes (CFTR, EDI and Bclx) [42–44] and the proportion of mRNA isoforms derived from each of them was analyzed by RT-PCR and quantified as indicated above. Different concentrations of the NS5 expression construct were co-transfected with each minigene. A dose dependent modulation of minigene-derived mRNA isoforms was observed upon expression of NS5 protein (Fig 4A). A quantitative analysis of PCR products corresponding to each isoform indicated 2, 5 and 1.5 fold change in the inclusion/exclusion ratios for CFTR, EDI and Bclx minigenes, respectively, in the presence of the viral protein (Fig 4B). The magnitude of the changes produced by NS5 on the analyzed splicing events is comparable to that observed when cellular levels of well-known splicing regulators were perturbed [45], indicating that NS5 modulates cellular splicing patterns.

NS5 exerts two enzymatic activities on RNA molecules, RNA polymerase and methyltransferase (MTase). In addition to acting on specific viral RNA templates, NS5 has non-specific terminal transferase activity, which adds non-templated nucleotides at the 3’ end of an RNA and it is capable of internally methylating RNA molecules [46,47]. Any of these activities could presumably modulate splicing, by altering pre-mRNA or snRNA properties. Thus, we evaluated whether the enzymatic activities of NS5 were involved in splicing modulation. To this end, we designed NS5 constructs with mutations that specifically impair either the MTase (Mut-MTase) or the RNA dependent RNA polymerase (Mut-RdRp) activity. Each of these mutant proteins retained the other (unmodified) enzymatic activity [47–49]. Using the minigene splicing reporter assay, it was found that both NS5 mutants were capable of causing a significant alteration of minigene alternative splicing patterns as compared with GFP or a control empty vector. The magnitude of the change observed with the two mutants was similar to that with the wild type NS5 protein (Fig 4C). These results suggest that NS5 binding to spliceosome components, but not its enzymatic activities, is required for splicing regulation.

In addition, we examined the impact of each domain of NS5 separately, by expressing the MTase or the RdRp domain, and only the RdRp showed a reduction of the inclusion/exclusion ratio of reporter minigenes (S4 Fig).

### Stability of snRNP complexes in infected cells

To investigate mechanistic aspects of the alteration of splicing by DENV infection and its association to NS5, we examined known properties of this viral protein. It has been previously reported that NS5, as a proteolitically processed protein originated from an uncleaved precursor, has the ability to induce STAT2 degradation by gathering the ubiquitin ligase UBR4 and STAT2 [21]. Based on this well established activity of NS5, we investigated whether DENV infection could alter the stability of splicing factors that were identified as NS5 binders. To examine this possibility, the amount of different spliceosomal proteins was analyzed in DENV infected and mock infected cells.

**Fig 5:**
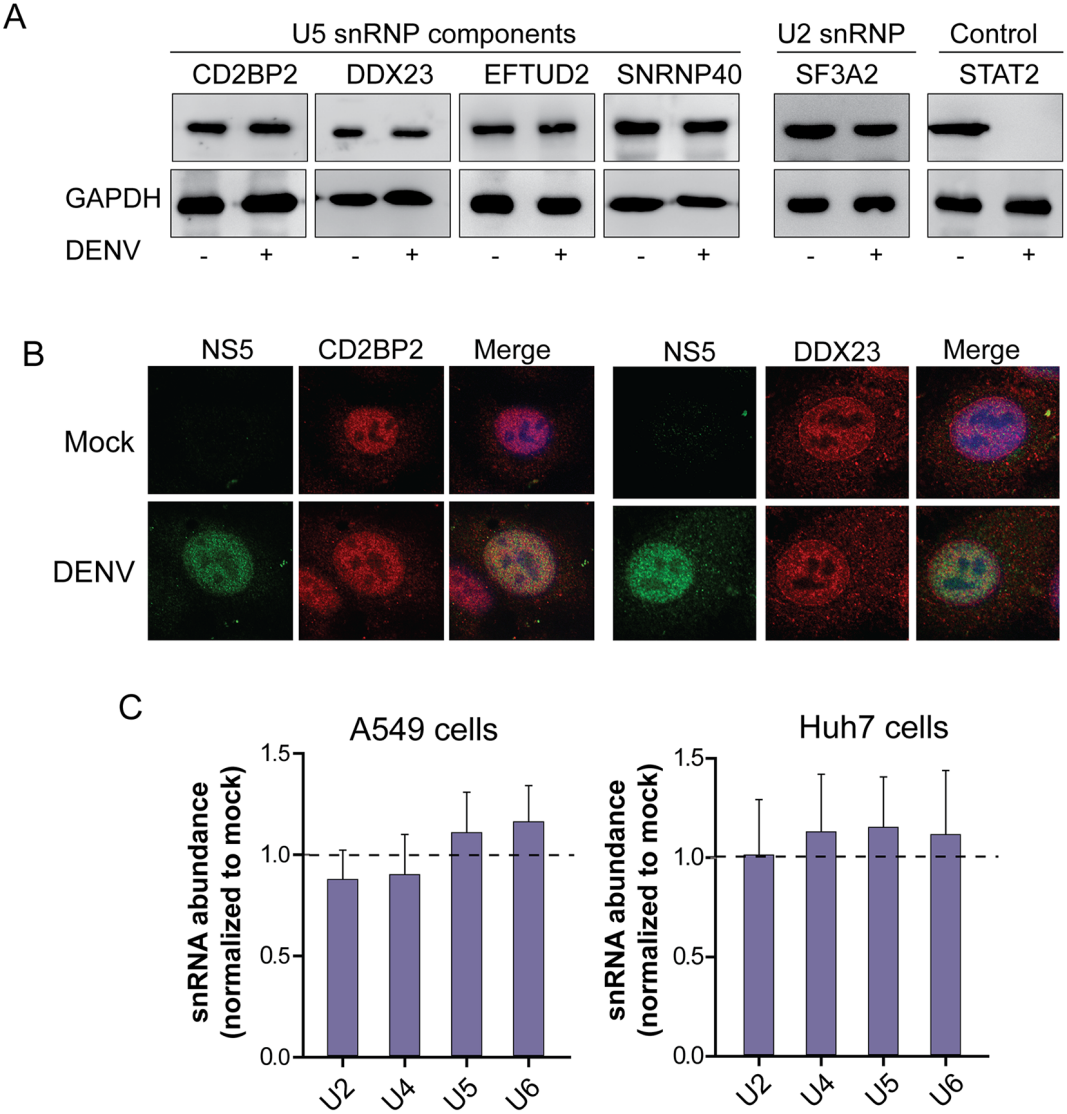
Abundance and localization of spliceosomal factors during DENV infection. (A) Western blots showing the levels of splicing proteins CD2BP2, DDX23, EFTUD2, SNRNP40 or SF3A2 and STAT2 used as a control, at 48 h post-infection. (B) Nuclear localization of CD2BP2 or DDX23 and NS5 in DENV infected or mock infected cells. (C) Determination of relative abundance of U2, U4, U5, and U6 snRNAs in DENV infected or mock A549 and Huh-7 cells.

Western blot analyses were performed at different times post infection to evaluate the levels of U5 snRNP specific proteins. As previously reported, extracts from DENV infected cells showed a drastic reduction of STAT2 levels, used as a control (Fig 5A). In contrast, no significant changes were observed for CD2BP2, DDX23, EFTUD2, SNRNP40 (U5 snRNP components) or SF3A2 (U2 snRNP component) levels up to 48 hours of infection as compared with those in uninfected cells (Fig 5A). We conclude that DENV infection does not induce degradation of these splicing factors as it was previously observed for STAT2.

Regarding subcellular distribution of splicing factors, it has been previously reported that Poliovirus 2A protease affects the function of certain splicing factors and RNA-binding proteins by regulating their nuclear-cytoplasmic shuttling [50] [51]. Thus, we evaluated a possible alteration of the subcellular localization of splicing proteins during DENV infection. Using confocal microscopy, we observed that while DENV2 NS5 protein localizes in the nucleus of infected cells, as previously described, viral infection was not associated to changes in the nuclear localization of spliceosomal factors. In this regard, NS5 as well as the U5 snRNP components, CD2BP2 and DDX23, were detected in the nucleus of infected cells (Fig 5B).

We then considered the possibility that NS5 binding to spliceosomal proteins could alter snRNPs organization and/or stability. It has been previously reported that a sensitive indicator for snRNP stability is the measurement of snRNAs abundance because these RNAs are unstable when freed from the RNP particles [52]. Thus, we measured the global relative amounts of U2, U4, U5, and U6 snRNAs by RT-qPCR in infected and uninfected cells. The results show no significant changes in snRNAs abundance during DENV infection in two different human cell lines (Fig 5C).

Together these data indicate that viral infection does not change the levels or the subcellular localization of U5 snRNP protein components, nor the stability of the snRNP complexes.

### NS5 interferes with the splicing reaction and reduces splicing efficiency of endogenous transcripts

**Fig 6:**
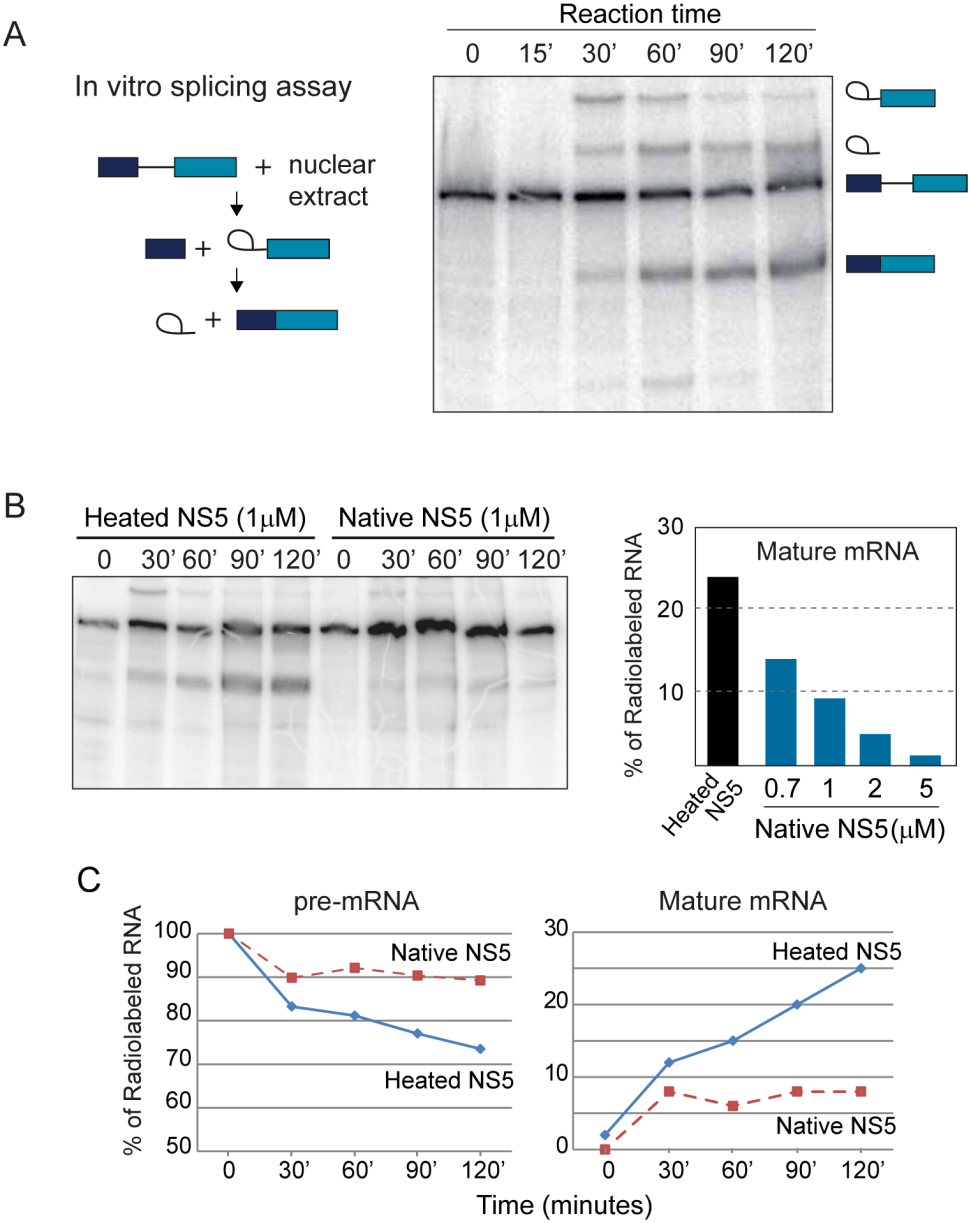
NS5 interferes with an in vitro splicing reaction and inhibits pre-mRNA maturation. (A) Time course of standard in vitro reaction showing radiolabeled substrate, intermediates and products of the splicing reaction. (B) Purified recombinant NS5 inhibits mature mRNA accumulation. A native or heat denature preparation of NS5 was added to the splicing reaction as indicated on the top of the gel. On the right, a graph showing dose-dependent inhibition of the splicing reaction by NS5. (C) Quantification of pre-mRNA and mature mRNA as a function of time in the presence of native NS5 or heated NS5 at 1μM.

To better understand the mechanism by which NS5 modulates splicing, we investigated whether NS5 has a direct effect on the splicing reaction. To examine this possibility, we used well-stablished in vitro system employing a radiolabeled pre-mRNA substrate that undergoes efficient splicing when combined with splicing-competent nuclear extracts from HeLa cells. This is a reconstituted system in which the substrate has been optimized to follow the reaction in controlled conditions [53–55]. The progress of the reaction over time can be monitored by denaturing polyacrylamide gel electrophoresis that allows the detection of initial substrates, RNA intermediates, and final splicing products, as exemplified in Fig 6A. The reaction was evaluated in the presence of purified NS5 protein, either using a heat-denatured or a native NS5 sample (Fig 6B). An unrelated protein was also used as control (S5 Fig). This study showed inhibition of mature mRNA product accumulation as well as lower amounts of intermediate (exon-lariat) when the native NS5 protein was present, suggesting inhibition of early catalytic steps. This is in agreement with sustained levels of pre-mRNA observed along the reaction in the presence of NS5 (Fig 6C). Also, addition of increasing concentrations of the viral protein to the reaction shows a dose dependent inhibitory effect on mature mRNA product accumulation (Fig 6B, right panel). These studies indicate that NS5 directly alters the efficiency of splicing by interfering at early stages of the reaction.

**Fig 7:**
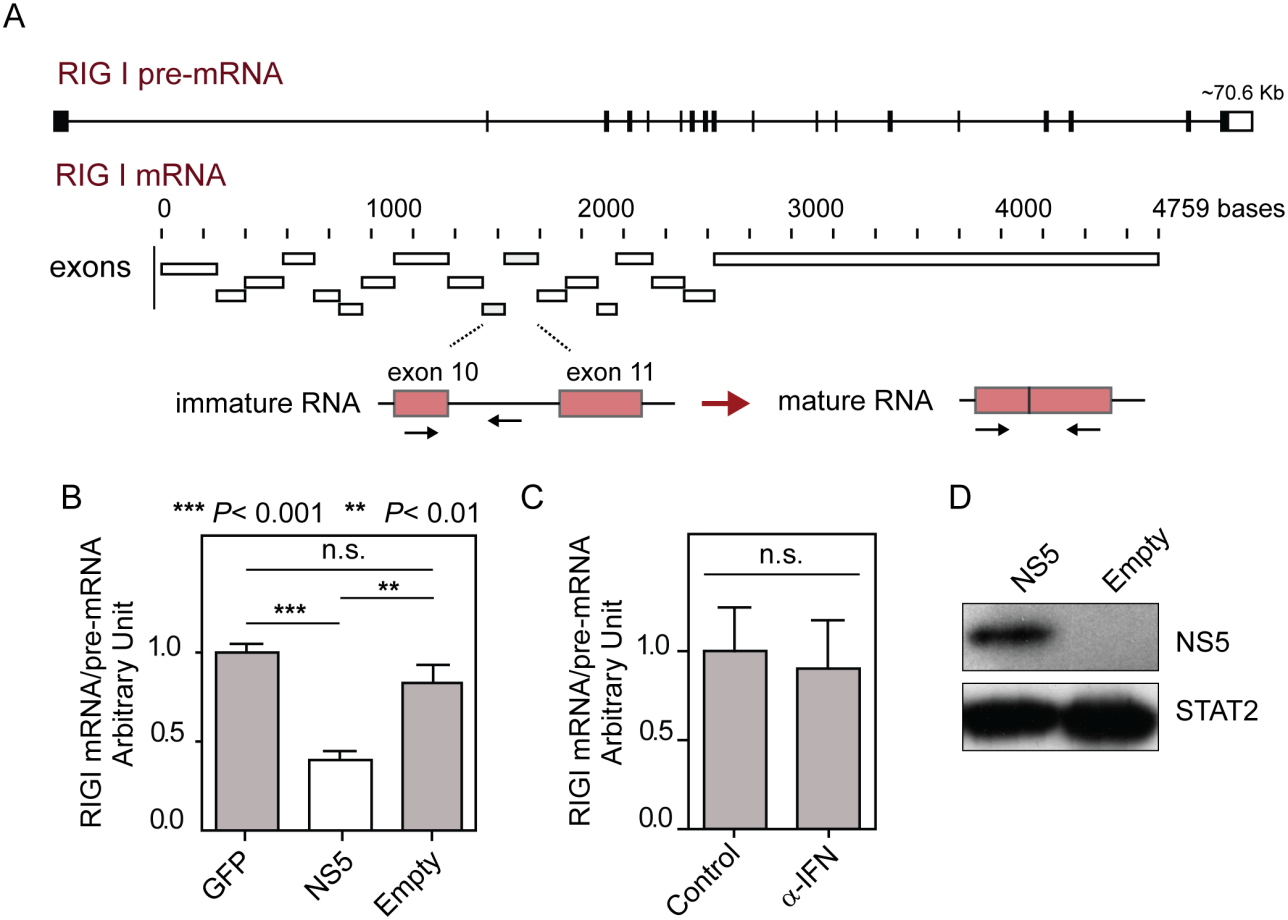
NS5 reduces the splicing efficiency of endogenous RIG-I mRNA. (A) Schematic representation of RIG-I pre-mRNA and mRNA. The abundance of these forms was measured by quantifying the immature and mature RNA transcripts using the region corresponding to exon 10, exon 11 and the intron between them, as indicated. The relative positions of the primers designed for RT-qPCR are depicted. (B) NS5 expressed as a mature protein significantly reduces the RIG-I splicing efficiency compared with GFP or an empty vector control. Data corresponding to four independent experiments is shown, mean ± SD. (C) RIG-I splicing efficiency (estimated as the RIG-I mRNA/pre-mRNA ratio) was not altered by α-IFN. (D) NS5 expressed as a mature protein does not induce STAT2 protein degradation.

It has been recently reported that RIG-I pre-mRNA splicing efficiency is susceptible to alterations in the EFTUD2 protein levels [30]. Considering that DENV NS5 interacts with this spliceosomal protein as shown in Fig 2A and interferes with an in vitro splicing reaction as shown if Fig 6, we analyzed the impact of NS5 on endogenous RIG-I pre-mRNA maturation. The RIG-I pre-mRNA is about 70 kb long and contains 18 exons. Based on this, we evaluated splicing efficiency by focusing at the excision of the intron between exons 10 and 11 (Fig 7A). To examine the influence of the mature NS5 protein on RIG-I mRNA processing, the ratio between mature- and pre-mRNA was evaluated by RT-qPCR. Interestingly, NS5 expression led to a significant reduction in RIG I splicing efficiency as compared with controls (p<0.001) (Fig 7B). The efficiency of RIG-I pre-mRNA maturation was found to be very robust under different conditions. For instance, RIG-I induction by α-IFN increased the levels of immature and mature forms of the transcript, and the efficiency of processing remained constant (Fig 7C), highlighting the effect observed with NS5. It is also worth noting that DENV NS5 inhibits IFN signaling by interfering with STAT2 as described above (Fig 4A). However, over-expression of mature NS5 is incapable of triggering STAT2 degradation [20] (Fig 7D). Together these results further support an impact of the viral protein NS5 on modulating splicing efficiency in vitro and in cell culture.

### Dengue virus infection reduces splicing efficiency

**Fig 8:**
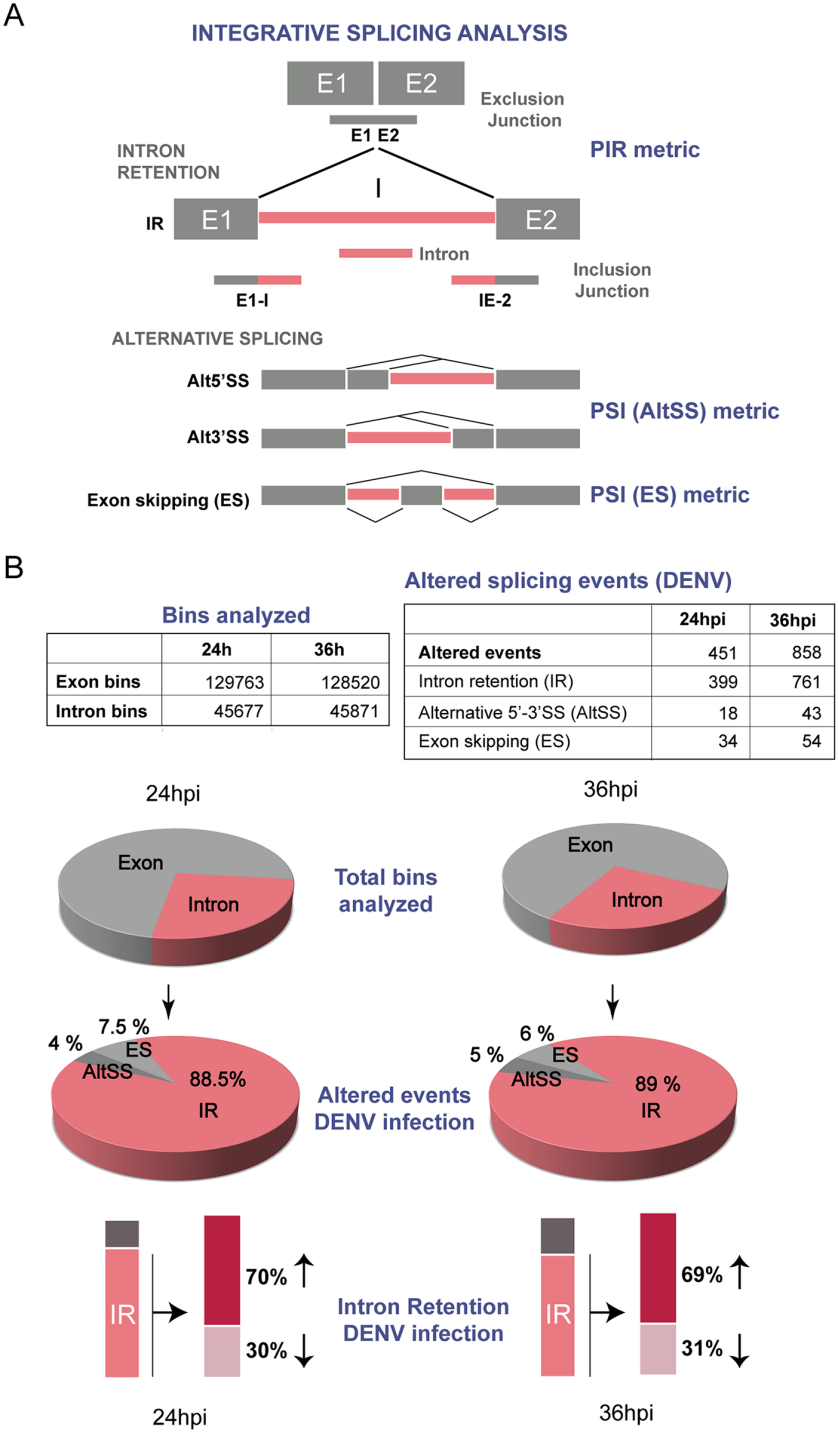
Decreased splicing efficiency in DENV infected cells. (A) Schematic representation of splicing analysis. The four splicing events analyzed are shown: Intron retention (IR), Exon skipping (ES), Alternative splice site donor/acceptor (Alt5’SS and Alt3’-SS). Constitutive exon and alternative 5’ and 3’SS regions are shown in grey and intron regions in pink. For intron retention the information of the junctions (E1-I, IE-2 and E1E2) was used to calculate the PIR metric. For ES and AltSS the PSI metric was used (see Materials and methods). (B) Data of splicing analysis of DENV or mock infected cells. On the upper panels, total amount of bins analyzed and altered splicing events for each time point post infection (24 and 36 hours). The percentage of altered events (ES, ALtSS and IR) is shown in pie charts. On the lower panel, increase or decrease retention of introns for each time point is shown.

To examine the global impact of DENV infection on splicing, we used genome wide RNA sequencing. Transcriptome analysis of DENV infected and mock infected cells at 24 and 36 hours post infection were performed, and a recently developed R package for an integrative splicing analysis was used [56]. ASpli combines statistical information from exon, intron, and splice junction differential usage to calculate differences in the percentage of exon inclusion (PSI metric) and percentage of intron retention (PIR metric) (Fig 8A and Materials and Methods). This method allows an unbiased evaluation of annotated and novel splicing events.

Biological triplicates of DENV and mock infected cells were used to construct mRNA libraries that were sequenced using an Illumina Hiseq 4000. The reads were mapped against the human genome (UCSC, HG19). For the analysis of differential splicing, multi-exonic genes were partitioned into features defined as “bins” corresponding to exonic regions, intronic regions and regions annotated as alternatively spliced. About 175000 bins were analyzed for each sample, 26% corresponding to intron bins and 74% to exon bins (Fig 8B). After the analysis, the data was filtered using a criteria to identify reliable changes in splicing during DENV infection. Using a threshold of difference in PSI or PIR between experimental conditions >5% and false discovery rate (FDR) of ≤10%, ASpli identified changes in 451 and 858 events at 24 and 36 hours post-infection, respectively (Fig 8B, S2 and S3 Tables). Interestingly, we observed an enrichment of intron sequences in mature RNAs at both 24 and 36 post DENV infection. Intron retention (IR) is the process by which an intron remains unspliced in a polyadenylated transcript, and although it was considered a rare event, increase IR rates have been recently reported in a large number of human cells [57]. In infected cells, IR increased in about 70% of the event that changed at 24 and 36 hours, indicating a reduction of intron excision in hundreds of transcripts, which could be associated to a perturbation of the splicing catalysis (Fig 8B) [57]. We conclude that DENV infection has an impact on splicing efficiency of a large subset of transcripts.

### Down regulation of U5 snRNP components enhances DENV replication

**Fig 9:**
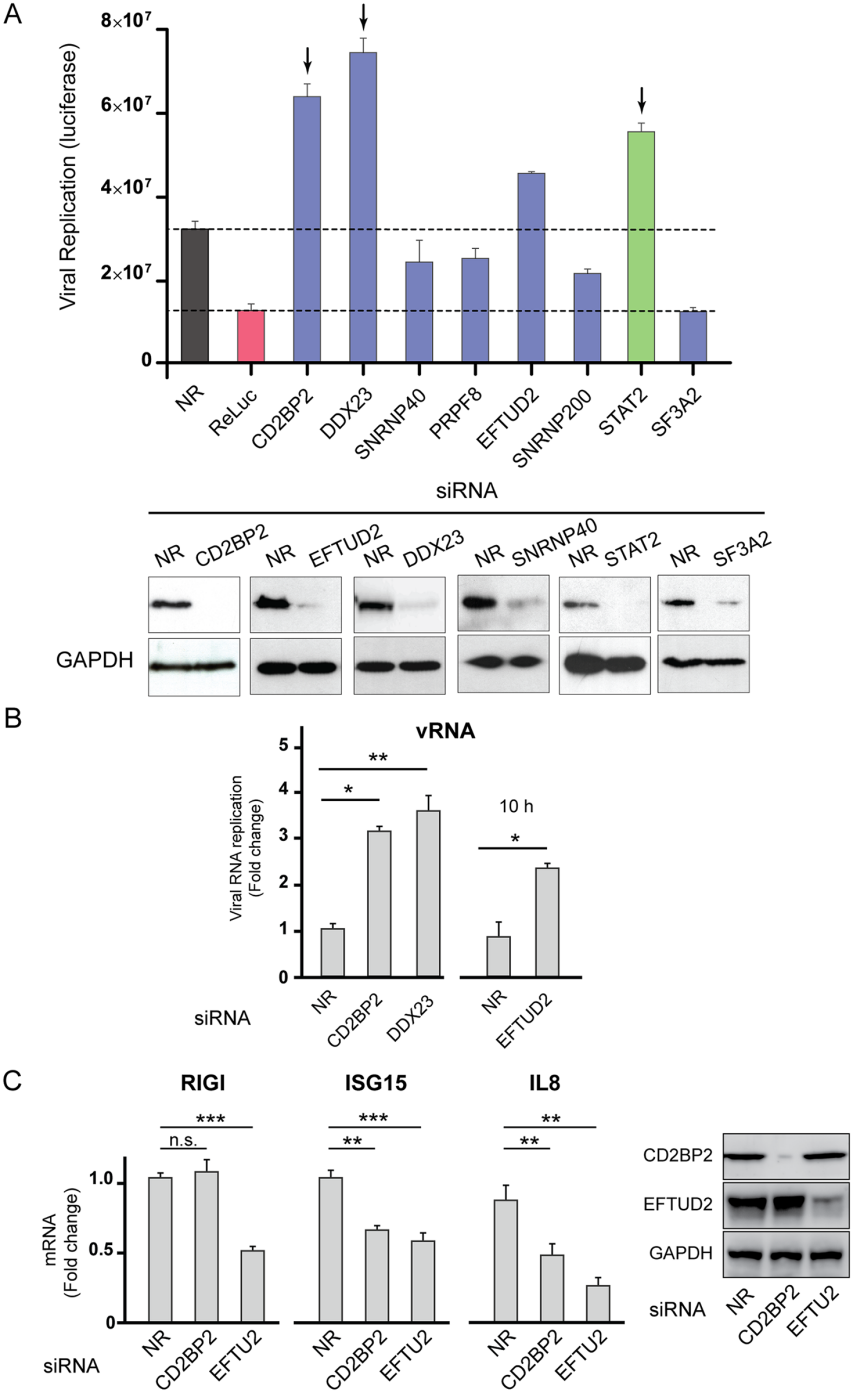
Silencing U5 snRNP components enhances DENV replication. (A) Reporter DENV replication in A549 cells transfected with siRNA directed to spliceosomal proteins (CD2BP2, DDX23, SNRNP40, PRPF8, EFTUD2, SNRNP200 or SF3A2). An infectious DENV carrying the luciferase gene was used. Three controls were included: a non-related siRNA (NR, black bar), siRNAs directed to Renilla luciferase (control for inhibition, red bar) and siRNA directed to STAT2 (control of NS5 binder with antiviral activity, green bar). Results are representative of three independent experiments (duplicates, mean ± SD). At the bottom, immunoblots indicate the levels of silenced proteins. (B) DENV replication in A549 cells transfected with a non-related siRNA (NR), or siRNAs directed to CD2BP2, DDX23 or EFTUD2, measured by quantitative real time PCR (mean ± SD) at 10hpi for EFTUD2 and 24hpi for CD2BP2 and DDX23. (C) Induced levels of mRNAs corresponding to RIGI, ISG15 and IL8 in A549 cells infected with NDV, which were previously silenced with siRNAs directed to CD2BP2 or EFTUD2 as indicated for each case. RNA levels were measured by quantitative real time PCR (mean ± SD). On the right, immunoblots indicate the levels of silenced proteins and loading controls.

Our proteomic data supported a novel interaction of DENV NS5 with U5 snRNP components, while in vitro and cell based assays indicated a role of the viral protein on modulating cellular splicing. In addition, transcriptome analysis of infected cells revealed splicing changes with intron removal perturbation in a large number of genes. To evaluate whether U5 snRNP composition is relevant for DENV infection, we conducted siRNA-mediated knockdowns of U5 proteins identified as NS5 binders and assessed the virus ability to infect and replicate (Fig 9). Specific siRNAs were transfected into A549 cells, 48 hours later the cells were infected with a reporter DENV virus encoding luciferase [58], and viral replication was evaluated as a function of time. Negative and positive controls, non-related siRNAs or siRNAs directed to the luciferase gene, respectively, were included. Silencing of STAT2 was used as a control for a negative regulator of viral replication that binds NS5. Interestingly, depletion of CD2BP2 or DDX23 showed a significant increase on viral replication. It is important to mention that the rise of viral replication detected upon silencing any of these U5 components was similar or higher than that obtained by silencing STAT2. In contrast to the effect observed with components of U5, silencing a core component of U2 snRNP (SF3A2) greatly reduced viral replication (Fig 9A), supporting the idea that viral modulation of cellular splicing must be specific to provide a beneficial environment for viral infection. To confirm this observation the impact of silencing core components of U5 on replication of the WT virus was evaluated. In this case, an increase in viral RNA replication was observed upon silencing CD2BP2, DDX23 or EFTUD2 (Fig 9B).

The favorable environment for viral replication observed upon silencing U5 components could be the consequence of changes in cellular splicing, including that of antiviral and/or proviral transcripts. To evaluate whether these splicing components modulate cellular innate immunity, we induced an antiviral state by infection with an unrelated virus that lacks known counteracting mechanisms (Newcastle disease virus. NDV) and evaluated gene expression of antiviral factors in cells that were or not silenced for U5 components (Fig 9C). Silencing EFTUD2 resulted in a significant reduction of RIGI, ISG15, and IL-8 induction, while knocking down CD2BP2 reduced the expression of ISG15 and IL-8. This observation provides a link between the splicing machinery, DENV infection and the host antiviral response, emphasizing the relevance of certain U5 components in restricting viral infection.

## Discussion

Here, we discovered a novel property of the DENV NS5 protein in subverting cellular splicing. Proteomic analysis and functional studies revealed that NS5 binds to active spliceosomes in infected cells and modulates splicing. Mechanistic studies indicate that NS5 interacts with components of the U5 snRNP and reduces the efficiency of pre-mRNA splicing. Our findings support a model in which NS5-mediated regulation of specific spliceosomal components renders an advantageous cellular environment for DENV replication, providing a new function for the viral NS5 polymerase in infected cells.

In this work, we present a comprehensive DENV NS5 protein-protein interaction network using a tagged infectious virus. The study revealed a significant enrichment of splicing related proteins, with particular abundance of U5 snRNP components. About 20% of the proteins identified were previously associated with NS5 or DENV infection. These proteins include STAT2, THRAP3, PRPF8, SNRNP200 and UBR4 that have been recently reported to interact with DENV NS5 [21,33], ERC1 that was found to be downregulated during DENV infection [59], and ILF3 that also interacts with the viral NS3 protein [60]. Because NS5 was proposed to function as an adaptor for the ubiquitination system in association with UBR4, and the screen also identified UBR5 as NS5 binder, it will be interesting to further evaluate whether this ubiquitin ligase is involved in NS5-mediated proteasome degradation of specific host factors. In addition, our study identified two members of the Y box binding protein family, YBX2 and YBX3, as NS5 binders. In this regard, YBX1 is an alternative splicing regulator that has been previously associated to DENV replication [61].

Based on the enrichment of splicing factors as NS5 binders, we evaluated the impact of DENV infection on cellular splicing by analyzing first a panel of endogenous well-characterized alternative splicing events. A substantial change in inclusion/exclusion ratios was observed when comparing infected versus mock infected cells. Differential splicing during DENV infection was also examined at the wide genome level by high throughput RNA sequencing. This analysis revealed changes in splicing and defects in intron removal during viral infection (Fig 8). It is important to mention that increased intron retention was recently associated to pathological conditions [62,63], and in some of these cases mutations or alterations of core splicing factors were reported [64]. We hypothesize that perturbation of splicing efficiency during DENV infection is, at least partially, explained by NS5 interaction with core splicing factors.

Our findings are in agreement with a recent study using RNAseq in DENV infected cells [38]. In this particular report, a transcriptome comparison of cells infected with WT or an attenuated strain of DENV1 revealed important differences in splicing of transcripts involved in innate immune responses and cell cycle control [38]. The alteration in cellular splicing observed during DENV replication could be the result of a combination of processes that take place within infected cells. On one hand, it can be a consequence of the cellular response to infection, associated with cellular damage or to a cellular antiviral state. On the other hand, splicing alteration could be a viral mechanism to counteract the antiviral response or to favor gene expression of pro-viral factors. In this regard, there is an emerging theme that links viral infection with specific alteration of U5 snRNP components. Several lines of evidence obtained with different RNA viruses support the significance of this emerging link. In the case of picornaviruses, it has been shown recently that the viral polymerase 3D binds to PRPF8, a core components of U5, and blocks mRNA maturation, with the consequent accumulation of the lariat intermediate [65]. In addition, RIPseq analysis of 3D-PRPF8 complexes identified transcripts associated to cellular growth, proliferation and differentiation [65]. In the case of hepatitis C virus (HCV), a siRNA screen aimed to detect genes associated to the antiviral response revealed the involvement of three specific splicing factors: EFTUD2, PRPF8, and SNRNP200, which are all components of the U5 snRNP complex [37]. Further research with HCV showed that EFTUD2 contributes to the antiviral response through RIG-I/MDA5 pre-mRNA maturation and it was found that viral infection down-regulates EFTUD2, resulting in splicing alteration of antiviral factors [30]. Our results using DENV are consistent with these previous observations and contribute to the model that viral infection perturbs cellular splicing. Despite the similarity with that described for HCV, the underlying mechanism exploited by DENV seems to be different since viral infection does not involve a significant reduction of the abundance of U5 components or a reduction in the assembly of snRNP complexes (Figs 4 and 5). In addition, the impact on splicing efficiency during DENV infections, observed by increase intron retention in polyadenylated transcripts, also provides an evidence for viral manipulation of the splicing machinery (Fig 8).

Mechanistic studies suggest that DENV NS5 binding to active spliceosomes modulates splicing efficiency, without involving any known NS5 enzymatic activity neither altering localization/abundance of splicing components during DENV infection (Figs 4 and 5). In addition, our results support that NS5 interaction with the spliceosome is not mediated by RNA binding because treatment with RNases did not impair the NS5-U5 complex interaction. In vitro studies using reconstituted systems with recombinant NS5 showed a clear reduction in the amount of RNA intermediates and pre-mRNA processing in the presence of the native NS5 protein. We propose a model in which binding of NS5 to protein components of the spliceosome reduces the efficiency of the splicing reaction. In the same line, evaluation of splicing efficiency of endogenous pre-mRNAs, such as RIG-I, as well as alternative splicing events derived from a variety of endogenous genes or reporter minigenes, showed that NS5 expression was sufficient to alter both splicing efficiency and alternative splicing. In this context, it is important to highlight that a growing body of experimental evidence points out that alterations in core spliceosomal components by depletion, mutation or pharmacological treatment, alter different cellular pathways. This seems to be dependent on the differential sensitivity or differential requirement for splicing factors for the processing of distinct pre-mRNAs [66,67].

Although NS5 alone is able to lower the efficiency of the splicing reaction in vitro and in cells, in the context of infection, splicing modulation is extremely more complex. In this regard, our findings with transcriptome analysis provide a new angle to study the link between splicing modulation and viral infection. The question is whether there are key changes in the splicing machinery and cellular gene expression that yields a more permissive scenario for viral replication.

Interestingly, manipulating the levels of core splicing factors resulted in splicing changes with different outcomes for viral infection. For instance, upon silencing certain U5 components we observed: a) increased DENV replication and b) reduced ability to induce the antiviral response by an unrelated virus. In contrast, silencing U2 components caused viral inhibition (Fig 9). This observation supports a model in which modulating specific splicing components leads to a more or less restrictive environment for viral replication. The finding that several RNA viruses evolved mechanisms to interfere with the U5 snRNP and that different viral proteins bind components of U5, points to a function of this specific splicing particle in facilitating an antiviral state and is consistent with the known essential roles of U5 components during spliceosome catalytic activation. In this context, our study provides novel information strengthening this emerging viral mechanism that manipulates cellular splicing.

DENV is currently the most significant insect-borne viral pathogen around the world without effective vaccines or antivirals. Ultimately, increased understanding of the host-virus interaction network at the molecular level will bring novel ideas for viral inhibition and control.

## Materials and Methods

### Cell lines

Mammalian cells: A549 cells (human lung adenocarcinoma epithelial cell line, ATCC, CCL-185) and Huh7 cells (human hepatoma cell line, JCRB Cell Bank #0403) were maintained in Dulbecco’s Modified Eagle’s Medium/ F-12 (Ham’s), HEK 293 cells (human embryonic kidney cell lines, ATCC CRL-1573) were maintained in Dulbecco’s Modified Eagle’s Medium, HeLa cells (human epithelioid cervix carcinoma cell line, ATCC, CCL2) ‎ were maintained in RPMI-1640 Medium, BHK-21 cells (baby hamster kidney cell line, ATCC, CCL-10) were maintained in Minimum Essential Medium Alpha. All the media were supplemented with 10% fetal bovine serum, 100 U/ml penicillin, and 100 μg/ml streptomycin. Mosquito cells: C6/36HT (*Aedes albopictus* cells ATCC, CRL-1660 were adapted to grow at 33°C) were maintained in Leibovitz's L-15 Medium, supplemented with 10% fetal bovine serum, 100 U/ml penicillin, 100 μg/ml streptomycin, 0.3% tryptose phosphate, 0.02% glutamine, and 1% MEM non-essential amino acids solution.

### DENV infectious clone and construction of recombinant viruses with Strep-tagged NS5

Wild-type virus was obtained from an infectious clone of the DENV2 strain 16681 (GenBank accession number U87411), plasmid pD2/ICAflII [68]. Recombinant viruses with a purification tag in NS5 (DENV NS5-tag) were generated by replacing sites *Nhe*I-*Nru*I (r1-DV), or *Stu*I-*Avr*II (r2-DV), or *Avr*II-*Afl*II (r3-DV and r4-DV) in pD2/ICAflII with fragments derived from overlapping PCRs containing the desired tag-coding sequence (a double Strep-tag sequence, amino acids WSHPQFEK-GGGS-WSHPQFEK).

### RNA transcription, transfection and viral stocks production

Viral RNA transcription and transfection was performed as previously described [69]. Briefly, DENV plasmids were linearized using *Xba*I and used as template for transcription with T7 RNA polymerase in the presence of cap analog (m7GpppA). RNA transcripts were transfected with Lipofectamine 2000 (Invitrogen) into BHK-21 or C6/36HT cells. Viral replication was assessed through indirect immunofluorescence. Supernatants were harvested at different times post-transfection and used to quantify infectious DENV particles by plaque assays.

### Antibodies

Rabbit polyclonal antibodies against CD2BP2, DDX23, EFTUD2, and SF3A2 were gently provided by Dr. Reinhard Lührmann (Max Planck Institute for Biophysical Chemistry, Gottingen, Germany). Antibodies against SNRNP40 (goat polyclonal) and against STAT2 (rabbit polyclonal) were purchased from Santa Cruz Biotechnology (sc-162407 and sc-476, respectively). Anti-Strep-tag antibody (mouse monoclonal) was purchased from EMD Millipore (71590–3). Anti-GAPDH (mouse monoclonal) was purchased from Abcam (ab8245). For the detection of the DENV envelope protein (E), mouse monoclonal anti-E antibody E18 was used [70]. Anti-NS5 antibodies (rabbit polyclonal and mouse polyclonal) were obtained in our laboratory.

### Immunofluorescence assays

Immunofluorescence assays were performed as previously described [71]. Briefly, A549 cells were seeded into 24-well plates containing glass coverslip. Twenty four hours later, the cells were mock infected or infected with DENV WT using a multiplicity of infection (MOI) of 10. After 24 hours, coverslips were collected and the cells were fixed with paraformaldehyde 4%, sucrose 4%, in PBS pH 7.4 at room temperature for 20 minutes. PFA fixed cells were then permeated with 0.1% Triton X-100 for 4 minutes at room temperature. Images were obtained with a Carl Zeiss LSM 5 Pascal confocal microscope.

### Purification of complexes containing Strep-tagged NS5 from DENV infected cells, human protein identification and construction of interaction network

Huh7 or HEK 293T (~2x10^7^) cells were either infected (MOI of ~1) with DENV WT, DENV NS5-tag or mock infected. At 48 hours post-infection, cells were harvested by scraping, washed with PBS, centrifuged at 100 rcf for 5 minutes at 4°C, and the pellet lysed with 1 ml of lysis buffer (50 mM Tris-HCl pH 7.4, 150 mM NaCl, 1 mM EDTA, 0.5% NP40, protease and phosphatase inhibitors, +/- DNase I and RNase A) chilled on ice. Cell lysates were incubated in rotating mixer for 30 minutes at 4°C and then clarified by centrifugation at 3000 rcf for 20 minutes at 4°C. The clarified lysates were over-night incubated with 30 μl Strep-tactin sepharose beads—50% suspension (IBA) in rotating mixer at 4°C. Beads were recovered by centrifugation at 400 rcf for 2 minutes at 4°C and washed four times with 1 ml of wash buffer (50 mM Tris-HCl pH 7.4, 150 mM NaCl, 1 mM EDTA, 0.05% NP40) chilled on ice. A fifth wash was conducted in absence of NP40 and beads were transferred to protein LoBind tubes (Eppendorf). Beads were incubated with 40 μl of elution buffer (50 mM Tris-HCl pH 7.4, 150 mM NaCl, 1 mM EDTA, 2.5 mM desthiobiotin) for 30 minutes at room temperature, continuous vortexing (beads in suspension). The eluates containing the purified protein complexes were obtained by a final centrifugation at 400 rcf for 2 minutes at 4°C and supernatant aspiration. Aliquots from the different samples were denatured in SDS sample buffer at 80°C for 15 minutes and loaded on precast 4–12% acrylamide gels (BioRad). Gels were stained with Pierce Silver Stain Kit (Thermo Scientific) or NOVEX Colloidal Blue Staining Kit (Invitrogen). Protein identification was conducted using a LTQ Orbitrap Velos (Thermo Scientific) mass spectrometer at the Department of Cellular and Molecular Pharmacology, University of California, San Francisco. STRING v10 ([72]; http://string-db.org) was used to retrieve the interactions among human proteins that co-purified with Strep-tagged NS5 and to determine enrichments for Gene Ontology (GO) categories.

### NS5 over-expression and pull-down

HEK 293T cells (~9x10^6^) were transfected using Lipofectamine 2000 (Invitrogen) with 12 μg of pcDNA NS5-Strep-tag or 1 μg pcDNA GFP-Strep-tag plus 11 μg empty pcDNA (as control). Cells were harvested 48 hours post-transfection by using a cell scraper, washed with PBS, and pelleted at 100 rcf for 5 minutes. Cell pellets were stored at -80°C before processing. The Strep-tag pull-down was conducted as described above, but introducing few modifications. Cells were lysed in 500 μl of lysis buffer, 10 μl Strep-tactin sepharose beads—50% suspension (IBA) were added to the clarified lysates, and after over-night incubation the beads were washed three times with 1 ml of wash buffer containing 0.5% NP40 plus once in absence of NP40. To analyze by SDS PAGE/Western blot the co-precipitation of different proteins, beads were heated for 10 min at 70°C in 50 μl of SDS sample buffer. Conversely, to analyze the presence of specific RNAs, beads were treated with 500 μl of Trizol (Invitrogen).

### Subcellular fractionation and NS5 distribution

A small-scale fractionation was conducted as described by [73]. Briefly, ~3 x10^6^ HEK 293T cells were transfected with 4 μg of pcDNA-NS5-Strep-tag or 0.4 μg pcDNA-GFP-Strep-tag plus 3.6 μg empty pcDNA (as control). After 48 hours, cells were harvested by using a cell scraper, washed with PBS, and pelleted at 100 rcf for 2 minutes. Cells were resuspended in 70 μl of Buffer A (10 mM HEPES pH 7.9, 10 mM KCl, 1.5 mM MgCl_2_, 0.34 M sucrose, 10% glycerol, 1 mM DTT, and protease inhibitors). Triton X-100 was added to a final concentration of 0.1%. Cells were incubated on ice for 8 minutes and then centrifuged at 1300 rcf for 5 minutes at 4°C. Supernatant (fraction S1) was separated from pellet (nuclei, fraction P1). Fraction S1 was clarified by centrifugation at 20000 rcf for 5 minutes at 4°C and the supernatant (cytoplasm, fraction S2) was collected. Fraction P1 was washed once with Buffer A, lysed for 30 minutes in 30 μl of Buffer B (3 mM EDTA, 0.2 mM EGTA, 1 mM DTT, and protease inhibitors), and centrifuged at 1700 rcf for 5 minutes at 4°C. Supernatant (nucleoplasm, fraction S3) was separated from pellet (chromatin, fraction P3). Fraction P3 was washed once with Buffer B. Fractions S2, S3, and P3 were diluted or resuspended in SDS sample buffer, heated for 10 min at 70°C, and analyzed by SDS PAGE/Western blot.

### Quantitative PCR for cellular RNAs (RT-qPCR)

Total cellular RNA was isolated by using Trizol (Invitrogen) and reverse transcribed to cDNA with random deca-oligonucleotide primer mix. Quantitative PCRs (qPCRs) were performed by using SYBR Green dye and specific primers for different snRNAs, pre-mRNAs, and mRNAs. GAPDH mRNA was used as a control housekeeping gene. The specific primers are listed in S4 Table.

### Analysis of endogenous alternative splicing events

Cells were mock infected or infected (MOI ~1) with DENV WT and harvested 48 hours post-infection. Total RNA was extracted by using Trizol (Invitrogen). The isolated RNA was subjected to RT-PCR in presence of α-^32^P dCTP. The specific primers are listed in S1 Table. Radiolabelled PCR products were electrophoresed in 6% polyacrylamide native gels, which were subsequently dried and exposed to X-ray films (Agfa). The bands detected by autoradigraphy were excised from gel and the radioactivity was measured in a scintillation counter (Cerenkov method) to then calculate the ratio between the amplification products corresponding to different mRNA isoforms.

### Assays with splicing reporter minigenes

The splicing reporter minigenes are described elsewhere [43,44,74]. HeLa cells (~0.5x10^6^) were transfected with 100 ng of each plasmid containing the indicated minigenes with different amounts of the empty pcDNA, pcDNA NS5-Strep-tag and/or pcDNA GFP-Strep-tag (total of 900 ng). Forty-eight hours later, RNA was extracted and subjected to RT-PCR in presence of α-^32^P dCTP. The ratio between the different splicing isoforms derived from each minigene was calculated as described above. NS5 polymerase and methyltransferease activity mutants were obtained by site-directed mutagenesis. For methyltransferase activity mutant the residue D146 was replaced by A [46,47] while for polymerase activity mutant the catalytic triplet GDD was replaced by AAA.

### In vitro splicing

MINX [^32^P]-labelled pre-mRNA was synthesized from a linearized MINX plasmid [54] using T7 RNA polymerase (Ambion). HeLa nuclear extract was prepared according to [55] and kindly provided by the Luhrmann laboratory. Splicing reactions were performed in the presence of 40% nuclear extract, 24 mM Hepes-KOH (pH 7.9), 2.4 mM MgCl_2_, 2mM ATP, 25 mM KCl and 20 mM creatine phosphate. Reactions were incubated at 30°C for the times indicated. RNA was extracted using Trizol and analyzed by 14% PAGE followed by autoradiography.

### cDNA library preparation and sequencing

Biological triplicates of DENV and mock infected cells were used for RNA-seq analysis. To construct the libraries, RNA was first extracted from cells using Trizol, followed by chloroform extraction and DNAse treatment and isopropanol precipitation. RNA quality was determined by 260/280 ratio using a Nanodrop 2000 and by agarose gel analysis of 28S and 18S rRNA bands. The resulting RNA was then PolyA purified using the BioO Scientific PolyA purification beads (catalog number 512979). PolyA purified RNA was then used to create sequencing libraries using the KAPA stranded RNA-seq kit (catalog number KK8400). Libraries were sequenced using paired-end reads on an Illumina Hiseq 4000.

### Differential high throughput splicing analysis

Sequence reads were aligned against the *Homo sapiens* genome (Hg19) with TopHat v2.1.1 [75] with default parameters. Count tables for the different feature levels were obtained from bam files using custom R scripts and considering the Hg19 transcriptome. Raw sequences (fastq files) and count tables at gene, exon, intron, AS bin and junction levels used in this paper have been deposited in the Gene Expression Omnibus (GEO) database (accession no. GSE84285).

We followed the strategy presented in ASpli [56] for the analysis of differential splicing. Briefly, multiexonic genes were partitioned into features defined as “bins” and then classified into exon, intron and annotated alternative splicing bins. Using this strategy, the transcriptome was partitioned in 468132 bins (S2 and S3 Tables). These datasets were then filtered according to several criteria applied at the gene and bin level. First, defined subgenic regions (i.e. bins) were considered for differential splicing analysis only if the genes with which they are associated were expressed above a minimum threshold level (more than 10 reads per gene and RD > 0.05) in all experimental conditions. Next, bins were considered for differential splicing analysis only if they had more than 5 reads and a RD bin/RD gene ratio > 0.05, in at least one experimental condition. After applying these filters, reads summarized at the bin level were normalized to the read counts of their corresponding gene. Differential bins usage was estimated using the edgeR package version 3.14.0 [76], and resulting *P*-values were adjusted using a false discovery rate (FDR) criterion. We then computed the metrics PSI (percent spliced-in) and PIR (percent intron retention), which were used as a filtering criteria for the splicing analysis combined with FDR (Fig 8). Bins with FDR lower than 0.1 and absolute delta PSI/PIR between 5 and 95% were considered differential used bins. The selected bins are included in supplementary information (S2 and S3 Tables).

### RNA interference DENV replication and induction of the antiviral response

RNA interference experiments were carried out using siGENOME ON-TARGET plus SMART pool siRNA oligonucleotides (Dharmacon RNA Technologies, Lafayette, CO, USA). The control non-related siRNA used was directed against Firefly luciferase. Twenty-four hours after seeding in 24-well plates, A549 cells were transfected with the corresponding siRNA using Oligofectamine (Invitrogen). Briefly, 25 pmol of siRNA in 50 μl of Opti-MEM (Invitrogen) were mixed with 2 μl of Oligofectamine in 50 μl of Opti-MEM and incubated for 20 minutes. The mix was added to a 50% confluent A549 cells monolayer and incubated overnight. Then, the supernatant was replaced with complete medium. After 48 hours of transfection, cells were infected with different viruses. For the reporter DENV containing the Renilla luciferase gene [71] cells were incubated for 36 hours prior to measuring luciferase activity. For infection with WT DENV (16681), cells were infected with a MOI of 10 and RNA was purified using Trizol at 10 and 24 hpi. For Newcastle diseade virus infection (NDV), transfected cells were infected with MOI of 0.1 and RNA was purified at 24 hpi. Quantitative real time PCR was performed as previously described using primers listed on S1 Table.

## Supporting Information

S1 Fig Replication properties of DENV containing a strep-tag fused to NS5. **A**. Immunofluorescence assays using specific anti-E antibodies showing replication of WT and NS5-strep (r4-DV) DENV at 1, 2 and 3 dpi. **B**. One-step growth curves of WT and r4-DV in BHK cells. The cells were infected at MOI of 0.01, and titers were determined at each time point by plaque assays. Error bars indicate standard errors of the means. **C**. Immunofluorescence assays using specific anti-NS5 antibodies showing nuclear localization of NS5 in WT and NS5-strep DENVs.
(TIF)

S2 Fig DENV infection modifies the splicing of host cells. Inclusion/exclusion ratios for DENV or mock infected cells for ZNF35 and Casp8 endogenous alternative exon-cassette events (duplicates, mean ± SD) in Huh-7 cells at 24 and 48hpi is shown.
(TIF)

S3 Fig DENV2 and DENV4 modifies host cell splicing differentially. Four events of exon cassette alternative splicing were evaluated in Huh7 cells at 48hpi. Representative autoradiographs and quantification of inclusion/exclusion ratio for each event are shown (triplicates, mean ± SD).
(TIF)

S4 Fig NS5 RdRp domain but not NS5 MTase domain interferes with alternative splicing. NS5 RdRp domain (Dom RdRp) alters splicing patterns from reporter mini-genes EDI and CFTR. Quantification of inclusion/exclusion ratio for each event is shown (mean ± SD).
(TIF)

S5 Fig *In vitro* splicing reaction. Time course of standard in vitro reaction is shown for an unrelated control protein (1 μg). Radiolabeled substrate, intermediates (free exon and exon-lariat) and products of the splicing reaction are observed.
(TIF)

S1 Table Individual dengue open reading frames corresponding to Capsid, NS3, NS4B and NS5 were cloned with a C-terminal 2xStreptavidin tag. Associated host proteins were identified by affinity purification and mass spectrometry. Data was analyzed using the MiST algorithm [77] which uses a linear combination of reproduciblity, specificity and abundance data to rank potential interactors with a score from 0 to 1. High confidence dengue-host pairs were considered when the MiST score was above 0.75. This cutoff was based on analysis of gold-standard HIV-host interactions [77]. MiST scores above the 0.75 cutoff are indicated. bt = below treshold, IB = immuno blot validated.
(XLSX)

S2 Table Data of splicing events altered during DENV infection at 24 hpi. Filtered differential bins according FDR (False Discovery Rate) and delta PSI/PIR (Percentage of Exon Inclusion/ Percentage of Intron Retention) metric
(XLSX)

S3 Table Data of splicing events altered during DENV infection at 36 hpi. Filtered differential bins according FDR (False Discovery Rate) and delta PSI/PIR (Percentage of Exon Inclusion/ Percentage of Intron Retention) metric
(XLSX)

S4 Table List of oligonucleotides used. (DOCX)
