# Supplementary figures and images for: The Dengue Virus NS5 Protein Intrudes in the Cellular Spliceosome and Modulates Splicing
Source: PLoS Pathog. 2016 Aug 30;12(8):e1005841. doi: 10.1371/journal.ppat.1005841 (PMC5004807; doi:10.1371/journal.ppat.1005841)

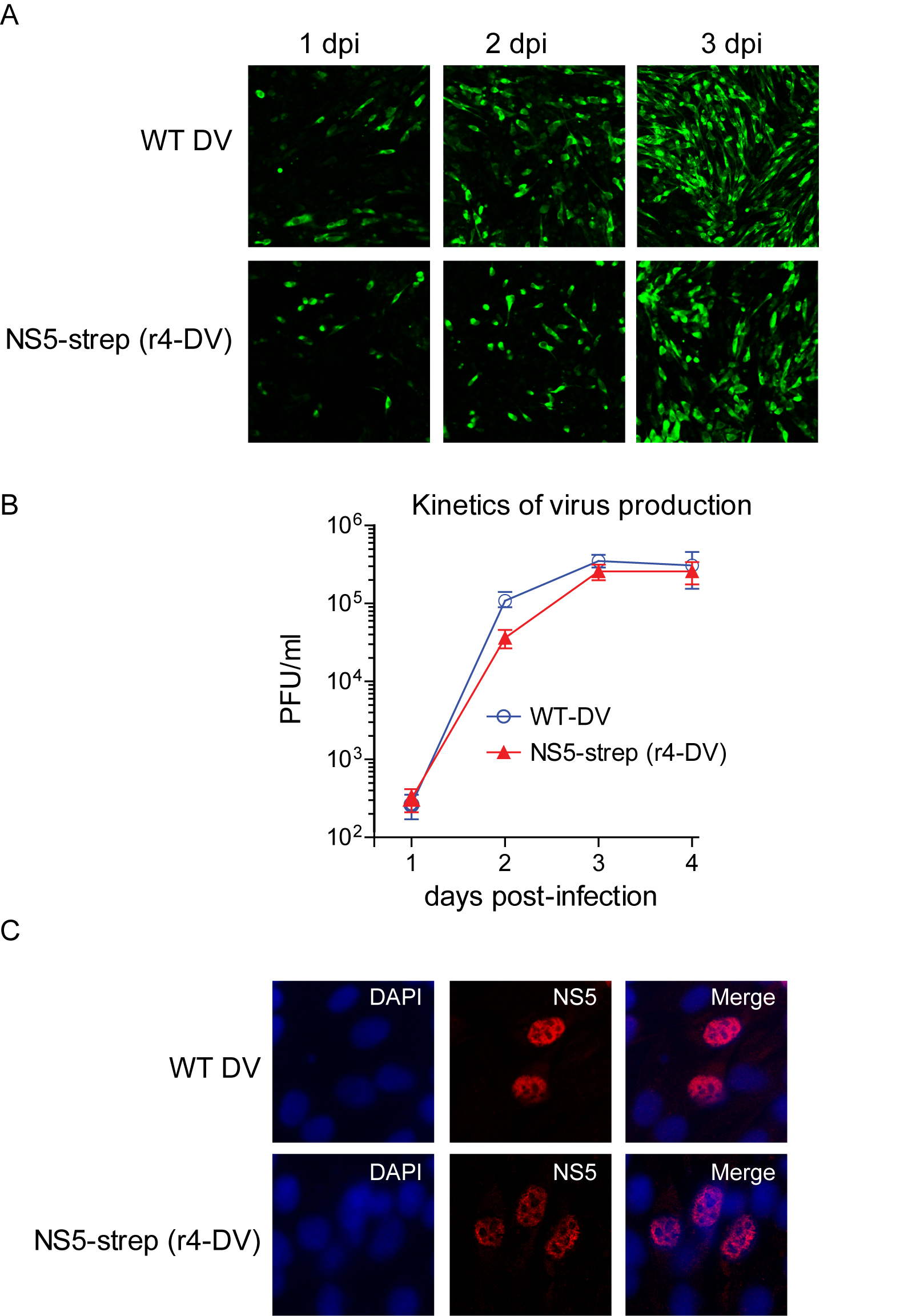

Supplement: S1 Fig — A. Immunofluorescence assays using specific anti-E antibodies showing replication of WT and NS5-strep (r4-DV) DENV at 1, 2 and 3 dpi. B. One-step growth curves of WT and r4-DV in BHK cells. The cells were infected at MOI of 0.01, and titers were determined at each time point by plaque assays. Error bars indicate standard errors of the means. C. Immunofluorescence assays using specific anti-NS5 antibodies showing nuclear localization of NS5 in WT and NS5-strep DENVs. (TIF) [file ppat.1005841.s001.tif]

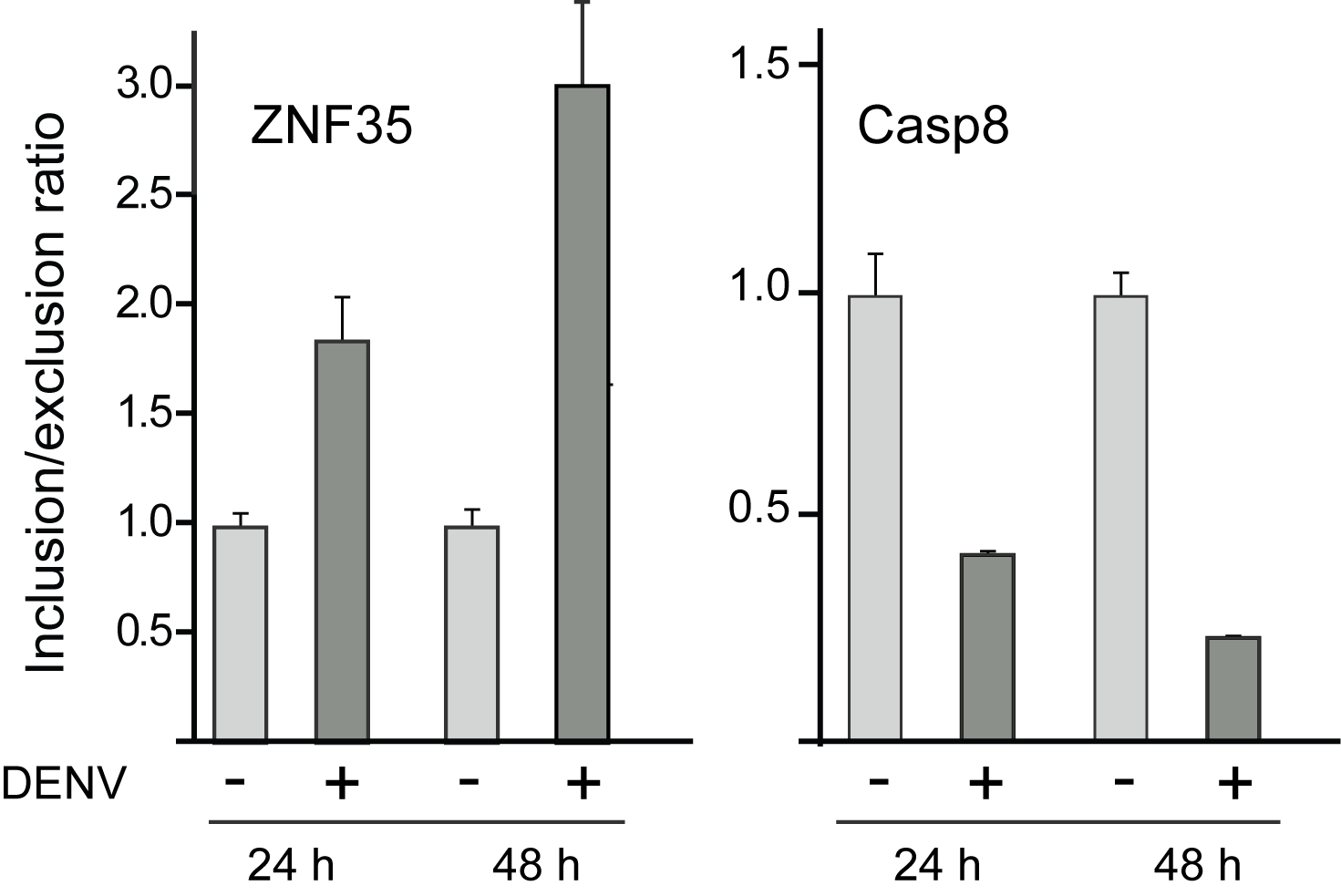

Supplement: S2 Fig — Inclusion/exclusion ratios for DENV or mock infected cells for ZNF35 and Casp8 endogenous alternative exon-cassette events (duplicates, mean ± SD) in Huh-7 cells at 24 and 48hpi is shown. (TIF) [file ppat.1005841.s002.tif]

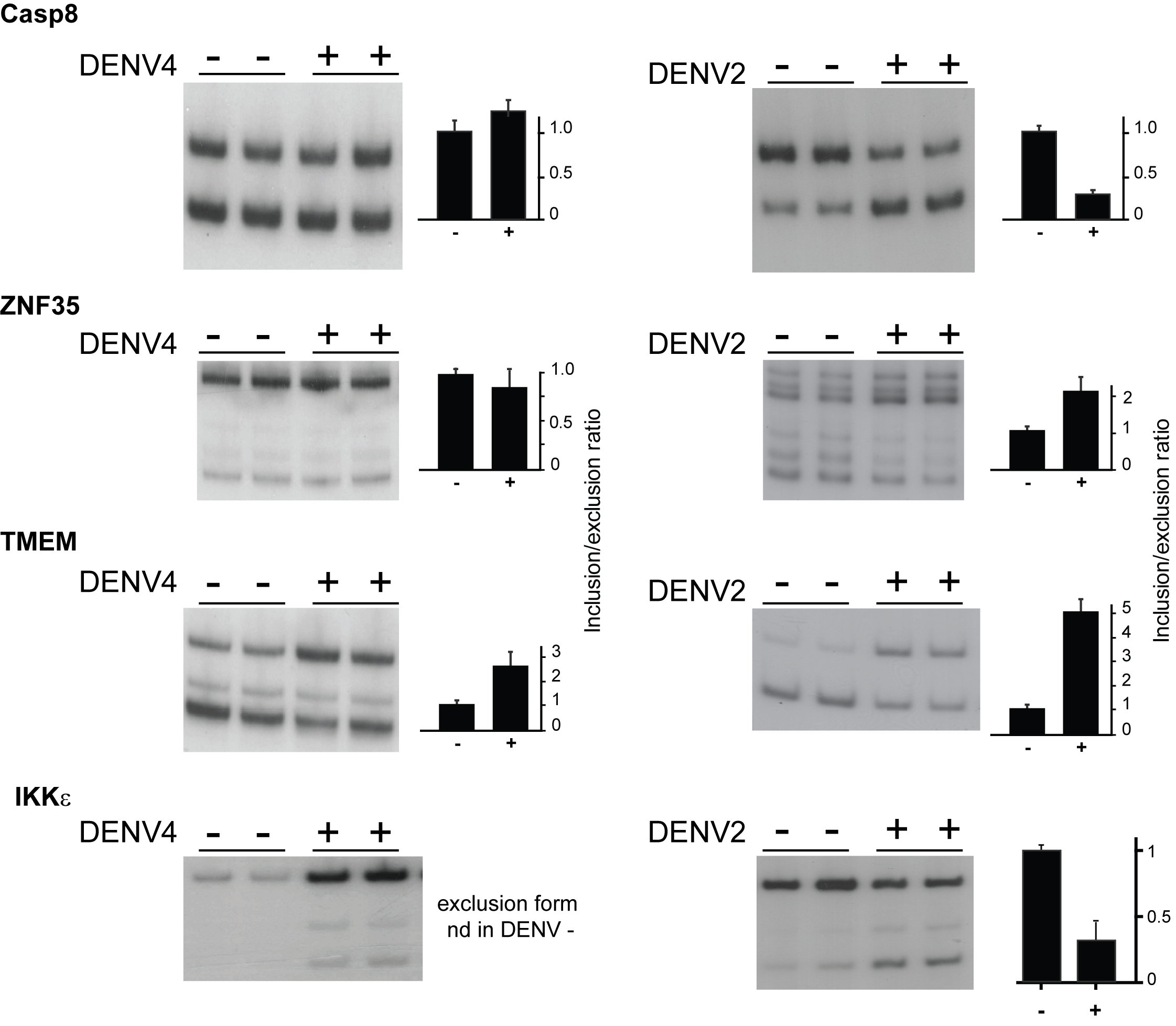

Supplement: S3 Fig — Four events of exon cassette alternative splicing were evaluated in Huh7 cells at 48hpi. Representative autoradiographs and quantification of inclusion/exclusion ratio for each event are shown (triplicates, mean ± SD). (TIF) [file ppat.1005841.s003.tif]

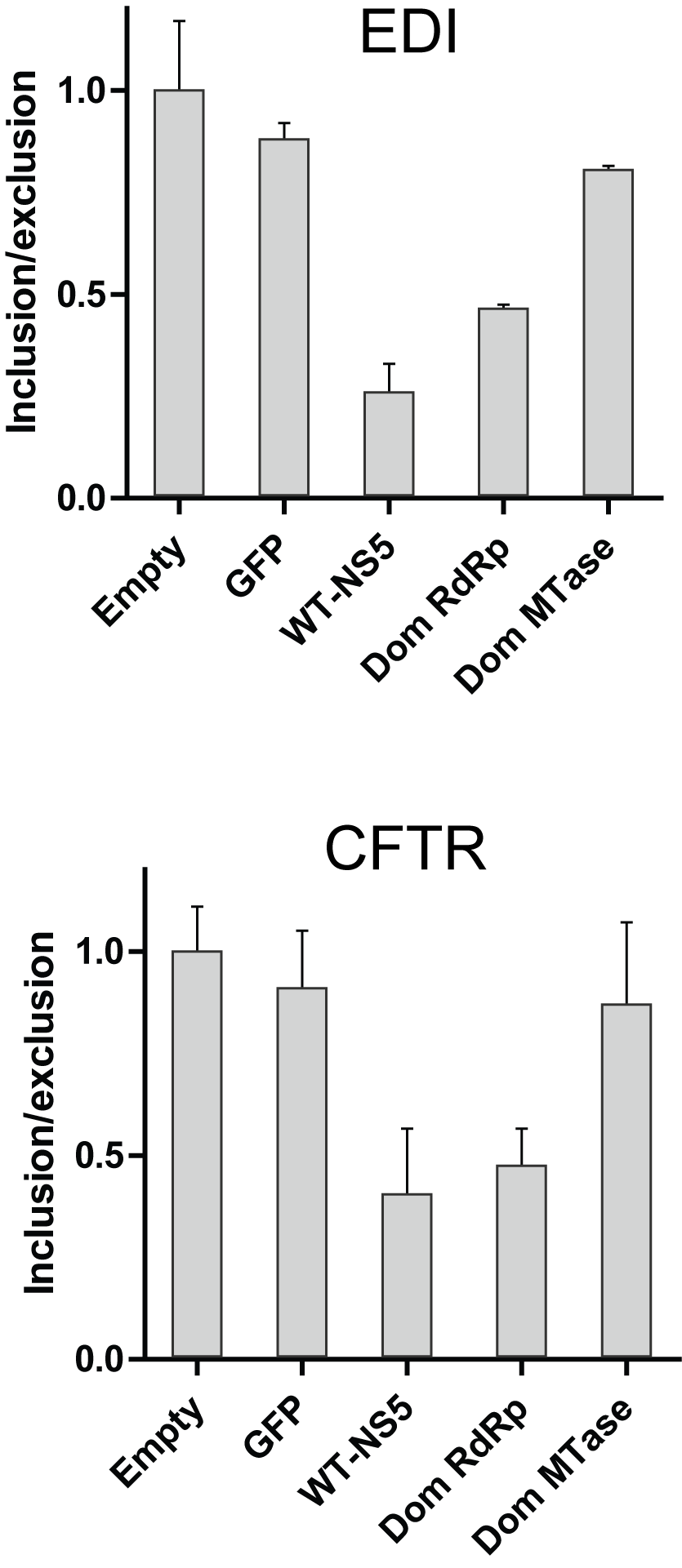

Supplement: S4 Fig — NS5 RdRp domain (Dom RdRp) alters splicing patterns from reporter mini-genes EDI and CFTR. Quantification of inclusion/exclusion ratio for each event is shown (mean ± SD). (TIF) [file ppat.1005841.s004.tif]

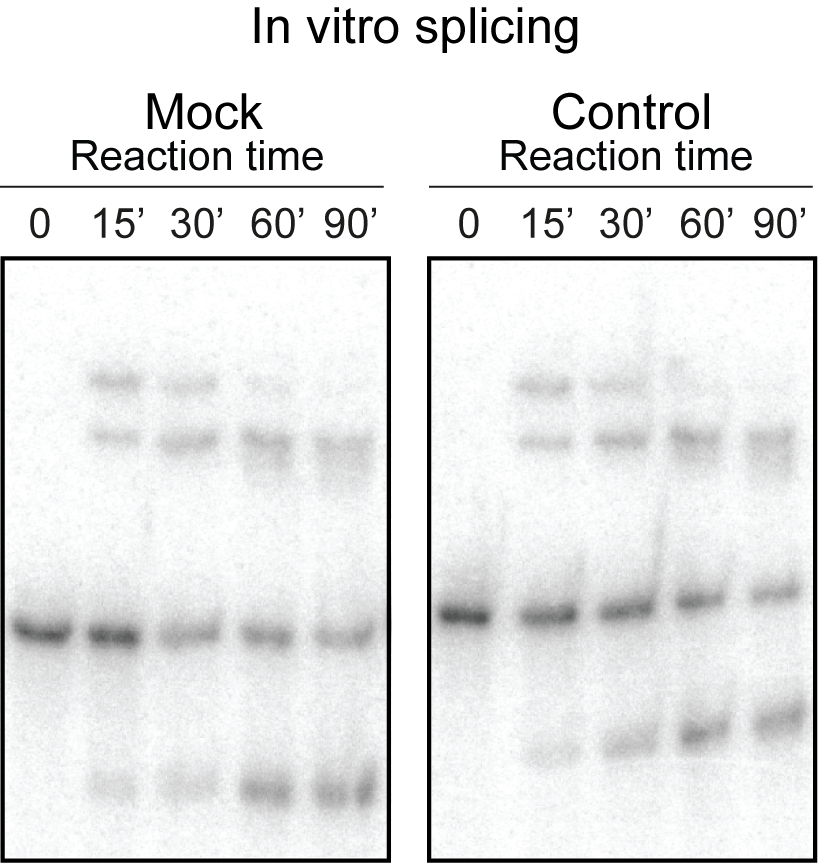

Supplement: S5 Fig — Time course of standard in vitro reaction is shown for an unrelated control protein (1 μg). Radiolabeled substrate, intermediates (free exon and exon-lariat) and products of the splicing reaction are observed. (TIF) [file ppat.1005841.s005.tif]
