# Supplementary material for: The Dengue Virus NS5 Protein Intrudes in the Cellular Spliceosome and Modulates Splicing
Source: PLoS Pathog. 2016 Aug 30;12(8):e1005841. doi: 10.1371/journal.ppat.1005841 (PMC5004807; doi:10.1371/journal.ppat.1005841)
Supplement: S4 Table — (DOCX) [file ppat.1005841.s009.docx]

**Table S4. List of oligonucleotides used**

**For RT-qPCR:**

|  | **Sense** | | **Antisense** | |  |
| --- | --- | --- | --- | --- | --- |
| **U1 snRNA** | CCATGATCACGAAGGTGGTTT | | ATGCAGTCGAGTTTCCCACAT | |  |
| **U2 snRNA** | TTCTCGGCCTTTTGGCTAAG | | CTCCCTGCTCCAAAAATCCA | |  |
| **U4 snRNA** | GCCAATGAGGTTTATCCGAGG | | TCAAAAATTGCCAATGCCG | |  |
| **U5 snRNA** | GGTTTCTCTTCAGATCGTATAAATC | | CTCAAAAAATTGGTTTAAGACTCAGA | |  |
| **U6 snRNA** | CTCGCTTCGGCAGCACA | | AACGCTTCACGAATTTGCGT | |  |
| **Akt pre-mRNA** | TGTCGCTGGCCCTAAGAAAC | | CTTGAGGAGGAAGTAGCGTGG | |  |
| **TBP pre-mRNA** | ACATGTGGTGTATGCAAATCCT | | CGTGGTTCGTGGCTCTCTTA | |  |
| **HSPCB pre-mRNA** | GGTACTTGGTGTGGCAAGGA | | AGGAACTGCAGCATTGGGTT | |  |
| **RPS9 pre-mRNA** | ACGGCGTCTGTTCGAAGGTG | | TGGAACAGAGGCAACAGAAGG | |  |
| **HPRT1 pre-mRNA** | AGCTAGCTAACTTCTCAAATCTTCT | | TTATGTCCCCTGTTGACTGGT | |  |
| **RIG-I mRNA** | GTGGAATCACGGATTAGCGACA | | TCTTTGTCTGGCATCTGGAACACC | |  |
| **RIG-I pre-mRNA** | GTGGAATCACGGATTAGCGACA | | CATTGTCTGAACTAAGGAGAACAC | |  |
| **IL8** | TTTTGCCAAGGAGTGCTAAAGA | | AACCCTCTGCACCCAGTTTTC | |  |
| **ISG15** | TCCTGGTGAGGAATAACAAGGG | | GTCAGCCAGAACAGGTCGTC | |  |
| **GAPDH** | CAATGACCCCTTCATTGACC | | GATCTCGCTCCTGGAAGATG | |  |
| **For RT-PCR** | |  | |  | |
| **FN1 (EDI)** | AGCCCCGCAAGCAGCAAGCC | | GTAGCATCTGTCACACGAG | |  |
| **EHBP1** | CAGCAAGATGAAGAGCGACGT | | AGTTACAGCTTTCTGGGCAGC | |  |
| **TMEM188** | GAAGCTGCGATGCGGACAG | | TTCTCCAGCGTCCAGTAGCAG | |  |
| **EED** | TGCGATGGTTAGGCGATTTG | | CCAAATGTCACACTGGCTGT | |  |
| **ZNF35** | TTGAGAGAAGCCATGGCCCTA | | GGGGTTTTTCAGCTTCCTCACA | |  |
| **PTBP2** | GCTGGTGGCAATACAGTCCTG | | TGGTTTCCATCAGCCATCTGT | |  |
| **Mcl1** | ATCTCTCGGTACCTTCGGGA | | AAATTAATGAATTCGGCGGG | |  |
| **Survivin** | GCATGGGTGCCCCGACGTTG | | GCTCCGGCCAGAGGCCTCAA | |  |
| **Apaf1** | CATCAGCAAATGAGAGGAAAA | | CCTCTGCAATCAGCCACCTT | |  |
| **Casp2** | GTTACCTGCACACCGAGTCACG | | GCGTGGTTCTTTCCATCTTGTTGGTCA | |  |
| **Casp8** | AACCTGCTGGATATTTTCATAG | | CTGTGATTCACTATCCTGTTC | |  |
| **Casp9** | GCTCTTCCTTTGTTCATCTCC | | CATCTGGCTCGGGGTTACTGC | |  |
| **IKBKE** | CCAGGAGAGTCTCAGCAAGC | | GGAGCTCAGACATCAGGAGG | |  |
| **MxA** | GCTGTTTACCAGACTCCGACA | | TGTAGGTGTCCTTGTCCTGC | |  |
